# Conferring DNA virus resistance with high specificity in plants using virus-inducible genome-editing system

**DOI:** 10.1186/s13059-018-1580-4

**Published:** 2018-11-15

**Authors:** Xiang Ji, Xiaomin Si, Yi Zhang, Huawei Zhang, Feng Zhang, Caixia Gao

**Affiliations:** 1 State Key Laboratory of Plant Cell and Chromosome Engineering, Center for Genome Editing, Institute of Genetics and Developmental Biology, Chinese Academy of Sciences, Beijing, China; 2 University of Chinese Academy of Sciences, Beijing, China; 3 Key Laboratory of Plant Stress Research, College of Life Science, Shandong Normal University, Jinan, China; 4 Department of Plant and Microbial Biology, University of Minnesota, Minneapolis, MN USA; 5 Center for Precision Plant Genomics, University of Minnesota, Minneapolis, MN USA

**Keywords:** CRISPR/Cas9 system, Geminivirus, Off-target, Virus-inducible, Virus-resistance

## Abstract

The CRISPR/Cas9 system has recently been engineered to confer resistance to geminiviruses in plants. However, we show here that the usefulness of this antiviral strategy is undermined by off-target effects identified by deep sequencing in *Arabidopsis*. We construct two virus-inducible CRISPR/Cas9 vectors that efficiently inhibit beet severe curly top virus (BSCTV) accumulation in both transient assays (*Nicotiana benthamiana*) and transgenic lines (*Arabidopsis*). Deep sequencing detects no off-target effect in candidate sites of the transgenic *Arabidopsis*. This kind of virus-inducible genome-editing system should be widely applicable for generating virus-resistant plants without off-target costs.

**Electronic supplementary material:**

The online version of this article (10.1186/s13059-018-1580-4) contains supplementary material, which is available to authorized users.

## Background

Agriculture worldwide is threatened by plant pathogens, such as plant viruses, which account for major losses in crop yields and revenues. For instance, it has been estimated that geminiviridae, a class of ssDNA viruses, could cause losses of billions of dollars to grain, vegetable, and fruit harvests every year. Thus, it has been highly desirable to develop plant varieties with improved geminiviruses resistance [1, 2]. Limited success was achieved through conventional transgenic approaches such as the use of pathogen-derived resistance (PDR) and RNA interference (RNAi) [3]. However, recent researches using the CRISPR/Cas9-mediated genome-editing technology showed promise to significantly reduce or even abolish disease symptoms in plants [4–8]. The CRISPR/Cas9 system originated in bacteria and archaea as an adaptive immune system and has been engineered to be a versatile genome-editing tool with applications in numerous organisms including targeting the genome of geminiviruses to inhibit their multiplication in plants. In these studies, Cas9 and single/multiple CRISPR guide RNAs were overexpressed to recognize and cleave the conserved sequences that are critical for geminivirus replication resulting in reduced virus load and symptoms [4–8]. However, one most important issue, namely the extent of off-target effects [9–11], has not yet been carefully examined in these CRISPR/Cas9-overexpression lines. Off-target effects usually occur due to tolerance of sgRNA sequence mismatches, and extended expression of Cas9 nuclease [12]. It has been shown that off-target effects could arise in mammalian cells even when there are as many as five mismatches with the sgRNA sequence especially in high dosages of Cas9 background [13–16]. Since off-target effects caused by CRISPR/Cas9 have raised regulatory concerns in both medical and agriculture applications, in this study, the extent of off-targeting was evaluated in virus-resistant plants that are overexpressing CRISPR/Cas9 constitutively. Furthermore, in order to mitigate the risk of off-targeting, a novel virus-inducible CRISPR/Cas9 system was developed to confer virus resistance with high efficiency and specificity.

## Results

**Fig. 1:**
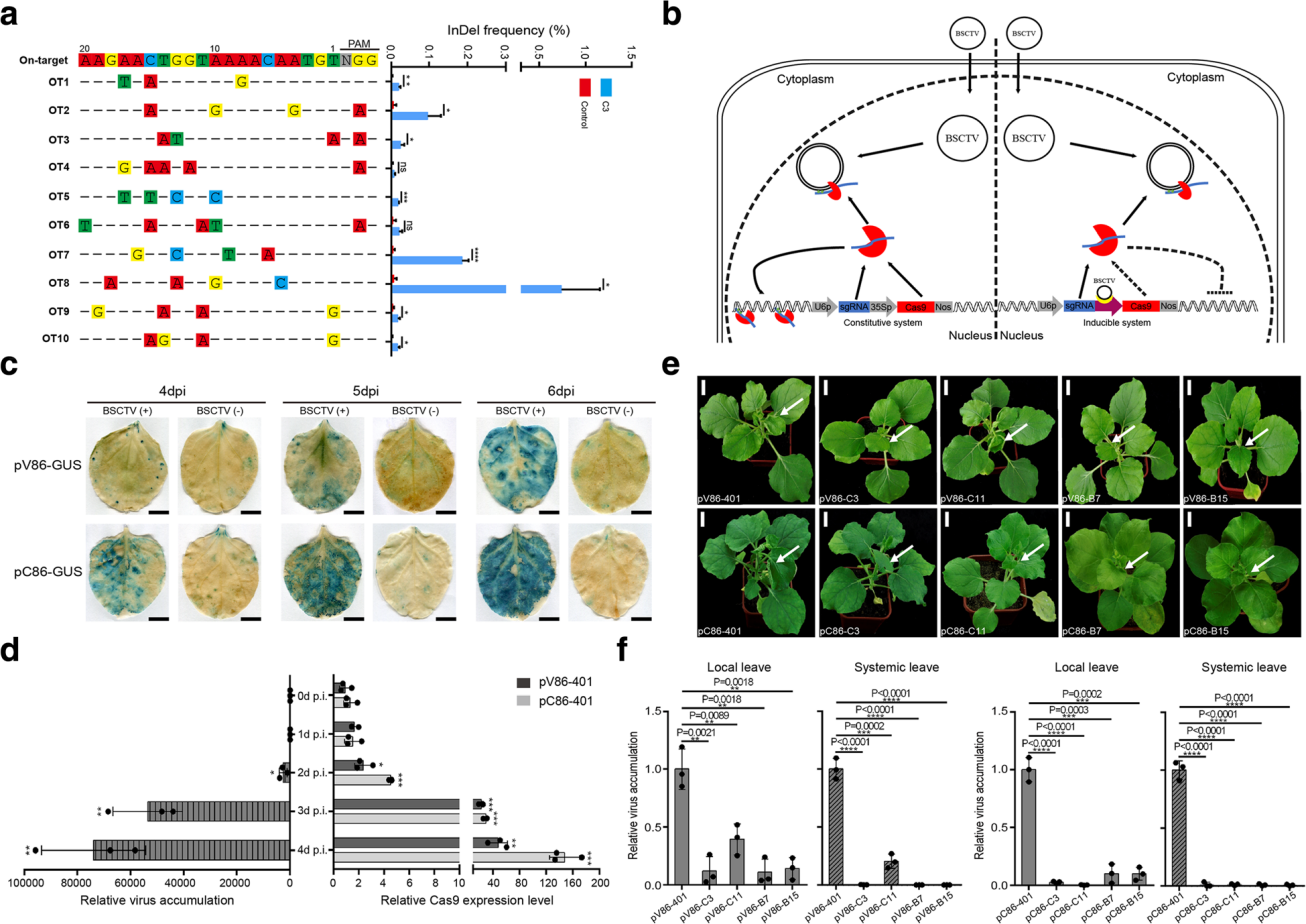
Off-target effects generated by overexpression of the CRISPR/Cas9 system, and validation of the VIGE system in transient assays based on *Nicotiana benthamiana*. **a** Off-target frequencies detected by deep sequencing in transgenic C3 and non-transgenic plants (*n* = 3). The genomic DNA of leaves was extracted for analysis. For each site, mismatches relative to the on-target site are shown by colored boxes, and bases in the spacer sequence are numbered from 1 (most PAM-proximal) to 20 (most PAM-distal). **b** Diagram of the virus inducible system that confers geminivirus resistance with high specificity. **c** Expression of the pV86-GUS and pC86-GUS reporter constructs. Leaves were stained with X-gluc 4, 5, and 6 days after virus infection. Scale bar, 1 cm. **d** Detection of BSCTV DNA accumulation level of individually expressed pCambia–BSCTV vector (left panel) and the Cas9 transcription level of inducible vectors (pV86-401 or pC86-401) co-expressed with pCambia–BSCTV (right panel) from 0 dpi to 4 dpi (*n* = 3). **e** Phenotypes reflecting the activities of the inducible systems (pV86-401 and pC86-401) containing B7, B15, C3, and C11 sgRNAs targeting the BSCTV genome in tobacco plants. White arrows indicate systemic leaves with altered phenotypes after BSCTV infection. Scale bar, 2 cm. **f** Virus loads in local and systemic leaves (*n* = 3) analyzed by quantitative PCR. Values are means ± S.D. *P < 0.05, **P < 0.01, ***P < 0.001, ****P < 0.0001; ns, no significant difference by two-tailed Student’s *t* test

To explore whether the constitutively overexpressed sgRNA/Cas9 complex induces off-target editing in host plants, we chose the T2 generation of virus-resistant *Arabidopsis* plants that harbored the constitutively overexpressed C3 sgRNA/Cas9 complex [6]. We searched for potential off-target sites of the C3 sgRNA sequence with Cas-OFFinder software [17] using either the canonical NGG or non-canonical NAG protospacer-associated motifs (PAMs) for SpCas9 in *Arabidopsis* and found that the minimal mismatches were 3 bp. We selected ten candidates with three or four mismatches, most of which were in the PAM distal region (Fig. 1a; Additional file 1: Table S1). Deep sequencing to detect the off-target events revealed that gene-editing events in the ten potential off-target sites in the host genome were significantly more frequent than in the controls (Fig. 1a; Additional file 1: Table S2). Of the ten candidate sites, eight had obvious off-target alterations, and off-target candidate 8 even had an average of 0.74% alterations (Fig. 1a; Additional file 1: Table S2). Thus, the overexpressed CRISPR/Cas9 system tends to induce off-target effects in the host plants.

It has been reported that inducible editing systems generate fewer off-target events [18, 19]. We therefore developed two novel virus-inducible CRISPR/Cas9 systems in which the expression of Cas9 is controlled in terms of location and time to reduce off-target effects (Fig. 1b), since the expression of intergenic regions (promoters) containing conserved *cis* elements in the geminiviruses genome can be inducibly regulated by the viruses themselves [20–22]. To construct a virus-inducible genome-editing (VIGE) system, we selected two beet severe curly top virus (BSCTV)-inducible promoters (pV86 and pC86) [20] and tested their inducibility in a transient system in *Nicotiana benthamiana* plants. The *GUS* gene driven by the pV86 or pC86 promoter was assembled into the pCambia1300 destination binary vector to generate constructs pV86-GUS and pC86-GUS (Additional file 1: Figures S1 and S2a). Mixed *Agrobacterium* cultures carrying pV86-GUS or pC86-GUS and pCambia–BSCTV clones were introduced together into *N. benthamiana* leaves by agroinoculation. *Agrobacterium* cultures harboring only pV86-GUS or pC86-GUS served as controls (Additional file 1: Figure S2b). From 4 days post infection (dpi) to 6 dpi, staining of leaves for GUS indicated that *GUS* expression driven by the pV86 promoter, and even more by the pC86 promoter, increased with time as the virus accumulated, while only very weak leaky expression was observed in the controls (Fig. 1c). These results indicated the pV86 and pC86 promoters were sensitive to induction by BSCTV and could be tunably trans-activated. Next, we constructed inducible CRISPR/Cas9 vectors (pV86-401 and pC86-401) with Cas9 driven by one or the other of the two BSCTV promoters and sgRNA driven by the AtU6 promoter (Additional file 1: Figure S3a). To investigate if the expression of Cas9 controlled by the two promoters could keep in pace with BSCTV accumulation, we transiently co-expressed the pV86-401 or pC86-401 vector with pCambia–BSCTV to detect the Cas9 transcription level and BSCTV accumulation in *N. benthamiana* leaves by agroinoculation. As shown in Fig. 1d, expression of Cas9 under both pV86 and pC86 promoters were significantly induced after BSCTV began to accumulate at 2 dpi, and pC86 promoter appeared to enable higher level induction than pV86 promoter. Thus, the inducible CRISPR/Cas9 vectors (pV86-401 and pC86-401) could rapidly response to BSCTV accumulation without delay.

To test the activities of the two systems against BSCTV, we next constructed pV86-sgRNA and pC86-sgRNA vectors using four highly active sgRNAs employed previously [6]: sgRNAs B7/B15 target the coat protein region and sgRNAs C3/C11 target the replication initiator protein region (Additional file 1: Figure S3b). We transiently expressed each pV86-sgRNA or pC86-sgRNA vector, with pV86-401 or pC86-401 as controls, in *N. benthamiana* leaves, and super-infected them with the pCambia–BSCTV construct by agroinoculation 2 days later. At 10 dpi, the systemic leaves of the control plants were stunted and curled (Fig. 1e), whereas the leaves of all the experimental plants were highly resistant to the BSCTV except those receiving sgRNA pV86-C11, which unexpectedly displayed mild symptoms of infection (Fig. 1e). When we quantified virus loads in both local and systemic leaves by qPCR analysis, we found that the virus loads in virus-resistant plants were significantly lower than in control plants (Fig. 1f; Additional file 1: Table S3). We next performed T7E1 assays to assess if these constructs had generated target mutations in the BSCTV in local and systemic leaves. Clear digested bands were found among amplicons amplified from the virus DNA in local leaves but not from the virus DNA in systemic leaves (Additional file 1: Figure S4a, b). Moreover, Sanger sequencing indicated that most of the mutations in the viruses from local leaves were deletions (Additional file 1: Figure S4a, b). Thus, we conclude that these virus-inducible CRISPR/Cas9 systems can target BSCTV efficiently.

In the above transient system, the inducer (BSCTV) was injected into leaves together with the inducible vectors. We next used our previous method [6] to inject separate *Agrobacterium* cultures containing the experimental vector, control vector (pV86-401 or pC86-401), and pCambia–BSCTV constructs into different regions of 30-day-old tobacco leaves (Additional file 1: Figure S5a). If the inducible CRISPR/Cas9 vectors were sufficiently sensitive to be activated when virus accumulated and migrated into the region containing the vectors, virus loads would be reduced. After 6 days, we quantified virus loads in regions containing a given experimental vector or control vector and found that all the experimental vectors inhibited virus accumulation by at least 42% (Additional file 1: Figure S5b, c and Table S4). Clearly, the inducible CRISPR/Cas9 system is effective in inhibiting virus replication.

**Fig. 2:**
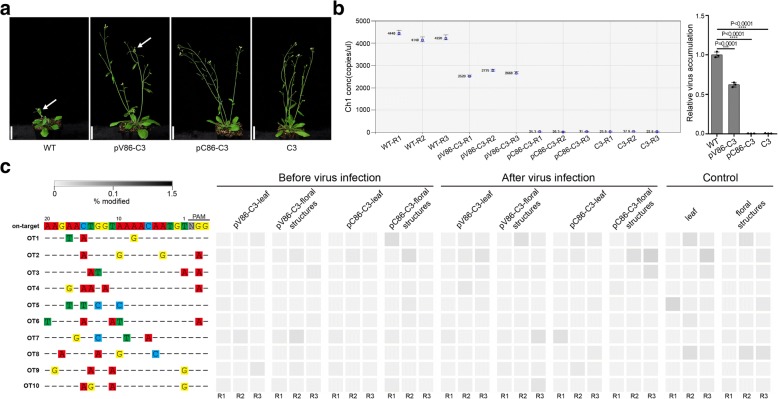
Transgenic *Arabidopsis* plants resistant to BSCTV have no off-target modifications at candidate sites. **a** Symptoms of non-transgenic and transgenic pV86-C3, pC86-C3, and C3 *Arabidopsis* plants after virus inoculation. Scale bar, 3 cm. White arrows indicate infected plants with symptoms. **b** Virus loads detected by ddPCR in non-transgenic and transgenic pV86-C3, pC86-C3, and C3 *Arabidopsis* plants after virus infection (*n* = 3). The left panel displays concentrations of virus copies as events per microliter, as automatically determined by the software. The right panel presents the relative virus accumulation in pV86-C3, pC86-C3, and C3 plants relative to control plants (*n* = 3). All values are means ± S.D. ***P < 0.001, ****P < 0.0001; two-tailed Student’s *t* test. **c** Indel frequencies at ten off-target candidate sites in transgenic pV86-C3, pC86-C3, and control plants determined by deep sequencing, presented as heat maps. Genomic DNA extracted from leaves and floral structures was analyzed. Each box in the heat map represents a single sequencing experiment of an individual plant. For each site, mismatches relative to the on-target site are shown by colored boxes, and bases in the spacer sequence are numbered from 1 (most PAM-proximal) to 20 (most PAM-distal)

We next assessed the anti-viral activity of the VIGE system in transgenic *Arabidopsis* plants. The pV86-C3 and pC86-C3 constructs were introduced into plants by *Agrobacterium*-mediated transformation. T2 generation plants were then infected with the virus. After 3 weeks, deformed floral structures and leaf curling were observed in the non-transgenic wild type plants (Fig. 2a). In contrast, the transgenic plants harboring the pC86-C3 construct showed strong virus-resistant phenotype similar to the positive control C3 plants that contained overexpressing Cas9 and C3 sgRNA construct [6]. The plants containing the pV86-C3 construct displayed mild symptoms consistent with the lesser induction level observed with this promoter (Fig. 2a). In order to measure the virus loads, we employed droplet digital PCR (ddPCR) [23, 24] to assess the levels of virus resistance in transgenic plants. Compared to the control plants, an average reduction of virus load of 41% relative to controls was detected in the transgenic pV86-C3 plants while virus accumulation was inhibited by over 99% in the transgenic pC86-C3 and C3 plants (Fig. 2b). These observations demonstrate that the VIGE system can efficiently increase virus resistance in transgenic plants.

Finally, we analyzed off-target effects in these transgenic plants. To explore whether the use of such an inducible system reduces off-target effects, and whether the trans-activated CRISPR/Cas9 system causes off-target effects in the act of defending viruses, we sequenced individual pV86-C3 and pC86-C3 plants before and after virus infection. We analyzed floral structures with inflorescences as well as leaves, to assess off-target effect over the entire plant. Deep sequencing did detect rare off-target modifications in transgenic plants both before and after virus infection together with non-transgenic plants (Fig. 2c and Additional file 1: Table S5), but statistical analysis showed that indel frequencies at the off-target sites in the pV86-C3 and pC86-C3 transgenic plants and non-transgenic controls did not differ (Additional file 1: Figure S6a, b). Thus, in contrast to the constitutively overexpressed CRISPR/Cas9 system, the virus-inducible system is highly specific.

## Discussion

Although the CRISPR/Cas9 system provides novel avenues for engineering plants resistant to DNA viruses, the problem of off-target effects in the plant genome could limit its utility. In this report, we showed that the constitutively overexpressed CRISPR/Cas9 system did indeed lead to off-target effects in host plants. To overcome this defect, we developed a virus-inducible CRISPR/Cas9 system. By using *GUS* reporters and detecting Cas9 expression level, we showed that the pV86 and pC86 promoters of BSCTV were tunably and rapidly trans-activated by co-infecting BSCTV so that CRISPR/Cas9 vectors driven by the pV86 and pC86 promoters were BSCTV-inducible; they were also found to be spatially and temporally responsive and to inhibit BSCTV accumulation in both transient (*N. benthamiana*) and transgenic (*Arabidopsis*) assays (Fig. 1e, f, Fig. 2a, b and Additional file 1: Figure S4, S5).

In a previous report, we noted a correlation between Cas9 expression level and anti-viral activity [6]. The lower expression driven by the pV86 promoter observed in the transient infection system (Fig. 1c, d), and previously [20], could account for that plants co-infected with the pV86-C3 vector and BSCTV showed low levels of virus resistance (Fig. 2a). This underlines the importance of choosing a strong virus-inducible promoter to create a highly efficient CRISPR/Cas9 system. When both pV86- and pC86-inducible systems were subjected to the off-target evaluation, no off-target effects were detected from the plants with either promoter even after excessive virus infections through the agrobacterium-mediated inoculation. This indicates the inducible CRISPR/Cas9 system significantly reduced off-target effects—the virus-inducible CRISPR/Cas9 system is activated quickly and is active for only a short time, thus decreasing the possibility of targeting the host genome, especially in uninfected cells.

Previous research has shown that virus-responsive promoters exist in a range of geminivirus genomes with the tight control under the viral proteins upon infection, and no evidence has been found on the induction of these promoters by other virus-unrelated conditions [20–22]. Thus, the virus inducible genome-editing system developed in this research should be widely applicable to defend plants against a variety of geminiviruses. This strategy could also decrease off-target mutations when multiplex sgRNAs are used to target several viruses. Recently, new CRISPR systems [25, 26], such as Cas13a, have been used to interfere with RNA viruses, and it should also be possible to create inducible systems that target RNA viruses.

## Conclusions

Our findings demonstrate that off-target effects are a constraint on constitutively overexpressed CRISPR/Cas9 systems conferring geminivirus resistance in plants. The virus-inducible CRISPR/Cas9 system that we have developed as an alternative is able to target and inhibit virus accumulation with no apparent off-target effects in the host genome, thus providing a novel, practical and effective tool for producing virus-resistant plants.

## Methods

### Plants materials

We used *Arabidopsis thaliana* ecotypes Col-0 and *N. benthamiana* in this study. T2 generation transgenic *Arabidopsis* plants, generated previously by the sgRNA C3 [6], were used for deep sequencing. Plant growth conditions and the production of transgenic *Arabidopsis thaliana* plants were as described [27].

### Deep amplicon sequencing

Genomic DNA of related transgenic and control plants was extracted and amplified with Phusion High-Fidelity DNA polymerase (New England Biolabs) using the primers listed in Additional file 1: Tables S2 and S5. PCR products were purified with an AxyPrep (Axygen) kit; their concentrations were normalized, and they were pooled into libraries, which were sequenced commercially (Mega Genomics, Beijing, China) by paired-end read sequencing using the Illumina NextSeq 500 platform. High-throughput sequencing data were analyzed with Cas-Analyzer [28].

### Construction of inducible-GUS reporter vectors, and staining of *GUS* activity in *N. benthamiana*

The pCambia-1300 binary vector was used as a backbone. A pCambia–BSCTV vector was used as template to amplify pV86 or pC86 trans-activated promoters using primers pV86-GUS-R1-F/R or pC86-GUS-R1-F/R (Additional file 1: Table S6). The GUS-Nost fragment was amplified with primers pV86-GUS-R2-F/R or pC86-GUS-R2-F/R (Additional file 1: Table S6), and pV86 or pC86 promoter fragments together with GUS-Nost were fused into XbaI-digested pCambia-1300 by the Gibson cloning method [29] using a ClonExpressII One Step Cloning Kit (Vazyme, Nanjing, China) to yield pV86-GUS and pC86-GUS vectors. The pV86-GUS, pC86-GUS, and pCambia1300-BSCTV vectors were individually transformed into *A. tumefaciens* strain EHA105. Cultures were transferred to LB medium with kanamycin and rifampicin overnight. After centrifugation, derivatives harboring the pV86-GUS or pC86-GUS vector were resuspended in 10 mM MgCl_2_ and 150 μM acetosyringone to a final OD_600_ of 2.0, while strains harboring pCambia1300-BSCTV were resuspended to a final OD_600_ of 0.5. One-milliliter samples of cultures of strains harboring the pV86-GUS or pC86-GUS vector were mixed with 1 ml of cells containing pCambia1300-BSCTV and the mixed suspensions were injected into true leaves of 1-month-old *N. benthamiana* plants with six to eight true leaves, using a 23-gauge needle. Leaves were harvested, incubated with X-gluc to localize GUS activity [30], and photographed with a digital scanner.

### Construction of the inducible CRISPR/Cas9 system and testing its activity in transient assays

The pHSN401 [31] vector as a backbone vector was digested with SphI and XbaI, and pCambia–BSCTV was used as a template to amplify pV86 or pC86 trans-activated promoters using primers pV86-401-F/R or pC86-401-F/R (Additional file 1: Table S6). The amplified promoter fragments were digested with SphI and XbaI and cloned into the backbone vector to generate the pV86-401 and pC86-401 inducible vectors. sgRNA oligos were introduced into pV86-401 and pC86-401 using the primers listed in Additional file 1: Table S6. Each construct together with a blank vector was introduced individually into *A. tumefaciens* strain EHA105. Transient assays of their activities were performed as described [6], and the T7E1 assays for detecting mutations followed the previously published protocol [32].

### Inoculation of BSCTV into transgenic *Arabidopsis*

*A. tumefaciens* strain GV3101 containing pCambia1300-BSCTV was grown at 28 °C in 2 ml LB broth with kanamycin and rifampicin to a final OD_600_ of 1.5, harvested by centrifugation, and resuspended to a final OD_600_ of 0.5. To infect *Arabidopsis* plants, we cut primary inflorescences when they were less than 1 cm in length and injected the cut stems with 1 ml of the suspension.

### RNA extraction, and RNA detection by qPCR

Total RNA was extracted with TRIzol (Invitrogen) then treated with RQ1 (RNA-Qualified) DNase I (Promega) and reverse transcribed into cDNA with SuperScript (Invitrogen). Cas9 transcription level was measured by qRT-PCR using SsoFast EvaGreen™ Supermix. *PPR* (pentatricopeptide repeat-containing protein) in *N. benthamiana* was used as an internal control.

### DNA extraction, and quantification of BSCTV accumulation by qPCR and ddPCR (droplet digital PCR)

Genomic DNA of *N. benthamiana* and *Arabidopsis* plants was extracted with cetyl trimethyl ammonium bromide (CTAB) buffer. *PPR* was used as the internal control for the qPCR assays.

A QX200 droplet digital PCR system (Bio-Rad) was used for droplet digital PCR. Genomic DNA samples of 2 μl (20 ng/μl) were added to 20-μl reaction mixes of BioRad QX200 EvaGreen supermix. The primers were present at final concentrations of 100 nM. The reaction mixes were briefly vortexed, avoiding the formation of bubbles. Each 20 μl reaction mix was loaded into a cell of a BioRad DG8 cartridge followed by addition of a 70-μl droplet generation oil. The cartridge was then placed into the droplet generator for droplet generation. Droplets were transferred to a 96-well PCR plate, heat-sealed with a pierceable foil seal, and PCR amplified. Amplification was conducted under the following standard cycling conditions: 95 °C for 5 min, followed by 40 cycles of 95 °C for 30 s; 60 °C for 60 s, 4 °C for 5 min, and 90 °C for 5 min, after which the plate was held at 4 °C; ramp rate 2 °C/s. Following PCR, the plate was put onto the QX200 Droplet Digital reader. Data were collected with Quantasoft™ Software.

## Additional file

Additional file 1: **Figure S1.** DNA sequences of the pV86 and pC86 promoters used in this study. Figure S2. Diagram of the transient system used to test the inducible activities of the pV86 and pC86 promoters. Figure S3. Constructs for the two BSCTV-inducible systems. Figure S4. Mutations induced in the BSCTV genome by the inducible pV86-401 (a) and pC86-401 (b) constructs. Figure S5. qPCR analysis of the sensitivity and efficiency of the inducible pV86-401 (b) and pC86-401 (c) combinations for inhibiting virus replication (*n* = 3) in *N. benthamiana* plants. Figure S6. Indel frequencies at ten candidate sites in the leaves (a) and floral structures (b) of transgenic pV86-C3 and pC86-C3 *Arabidopsis* plants were similar to the frequencies in non-transgenic control *Arabidopsis* plants (*n* = 3). Table S1. Potential off-target sites for C3 sgRNA in *Arabidopsis*. Table S2. Indel frequencies detected by deep sequencing of transgenic C3 and control *Arabidopsis* plants. Table S3. Relative levels of virus accumulation for each of the four target sites in the inducible systems. Table S4. The relative viral load detected from the experiment of inhibiting replicating virus. Table S5. InDel frequencies detected by deep sequencing with their primers in transgenic pV86-C3, pC86-C3, and control *Arabidopsis* plants. Table S6. Other primers used in this study. (PDF 2070 kb)
