# Supplementary material for: Conferring DNA virus resistance with high specificity in plants using virus-inducible genome-editing system
Source: Genome Biol. 2018 Nov 15;19:197. doi: 10.1186/s13059-018-1580-4 (PMC6238286; doi:10.1186/s13059-018-1580-4)
Supplement: Supplementary file 1 — Figure S1. DNA sequences of the pV86 and pC86 promoters used in this study. Figure S2. Diagram of the transient system used to test the inducible activities of the pV86 and pC86 promoters. Figure S3. Constructs for the two BSCTV-inducible systems. Figure S4. Mutations induced in the BSCTV genome by the inducible pV86-401 (a) and pC86-401 (b) constructs. Figure S5. qPCR analysis of the sensitivity and efficiency of the inducible pV86-401 (b) and pC86-401 (c) combinations for inhibiting virus replication (n = 3) in N. benthamiana plants. Figure S6. Indel frequencies at ten candidate sites in the leaves (a) and floral structures (b) of transgenic pV86-C3 and pC86-C3 Arabidopsis plants were similar to the frequencies in non-transgenic control Arabidopsis plants (n = 3). Table S1. Potential off-target sites for C3 sgRNA in Arabidopsis. Table S2. Indel frequencies detected by deep sequencing of transgenic C3 and control Arabidopsis plants. Table S3. Relative levels of virus accumulation for each of the four target sites in the inducible systems. Table S4. The relative viral load detected from the experiment of inhibiting replicating virus. Table S5. InDel frequencies detected by deep sequencing with their primers in transgenic pV86-C3, pC86-C3, and control Arabidopsis plants. Table S6. Other primers used in this study. (PDF 2070 kb) [file 13059_2018_1580_MOESM1_ESM.pdf]

a

pV86 promoter

ATTGAATCGGGCTCTCTTCAAATCCCCTATCAATTGGGTGCTTTGGGTGCTCTTAAATACCACCAAGGGGCC
ATCCGCATTAATATTACCGGATGGCCCCCAAAAAATACGTGGCCCAATGAAAATATGCCACGTGGAAAGCTA
AAGATGTTGATGTGATGGATTTGGGCGGGAAACTTCCTGAAGAAGTTTTCATTCCCGCCCCTTTTTTTGACT
TAGTCAAAAAAGTAGCCGACATACTGTTTGAAATAAAGTTAAAAGTAGATCATTGTGGTCCCCACAAGAAA
CTTGCTAAGCAAGTTTCGAATTATTTCTGTTTAAATACGTTATTTTTACATGTATATGTACTTATAAATGATGGT
CTGTCTACCAGACTGGTTATTTTTGCTATTTATCTTCAGTATTCTCCTGCAATCAGGTACGAACTTTTATGGGA
CCTTTCA

b

pC86 promoter

AGGAGCTGTTCTAAGGCGTCTTCTTTGGTTACTGAACATTGAGGGTATGTAAGGAAAAAATTTTTGGCTTT
TTTGTAAAAAGGCATATTGAATCGGGCTCTCTTCAAATCCCCTATCAATTGGGTGCTTTGGGTGCTCTTAAA
TACCACCAAGGGGCCATCCGCATTAATATTACCGGATGGCCCCCAAAAAATACGTGGCCCAATGAAAATAT
GCCACGTGGAAAGCTAAAGATGTTGATGTGATGGATTTGGGCGGGAAACTTCCTGAAGAAGTTTTCATTC
CCGCCCCTTTTTTTGACTTAGTCAAAAAAGTAGCCGACATACTGTTTGAAATAAAGTTAAAAGTAGATCAT
TGTGGTCCCCACAAGAAACTTGCTAAGCAAGTTTCGAATTATTTCTGTTTAAATACGTTATTTTTACATGTA
TATGTACTTATAAATG

Figure S1. DNA sequences of the pV86 and pC86 promoters used in this study. The pV86 promoter is colored in black (a) and the pC86 promoter in orange (b).

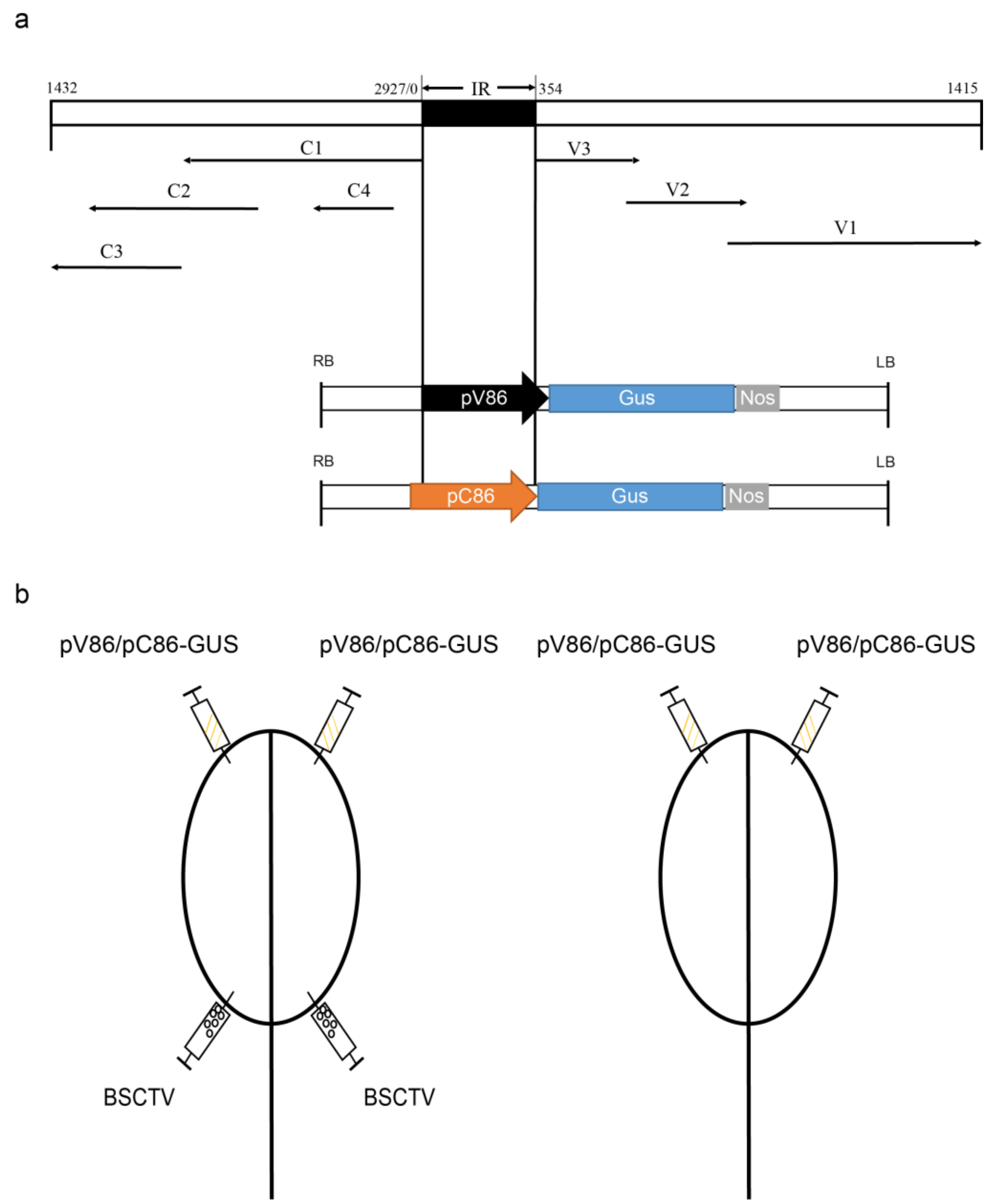

Figure S2. Diagram of the transient system used to test the inducible activities of the pV86 and pC86 promoters. (a) GUS reporter constructs based on the pV86 and pC86 promoters. The intergenic region of the BSCTV genome is indicated by a black box, and open boxes are coding regions. Open reading frames are indicated by solid arrows, and numbers indicate nucleotide positions in the BSCTV genomes. The pV86 promoter contains the intergenic region with an additional 86 bp of the V3 coding region while the pC86 promoter contains the intergenic region with an additional 86 bp of the 5' upstream region. (b) Strategy for testing the GUS reporter systems. Thirty-day-old *N. benthamiana* plants with between 6 and 8 true leaves were used.

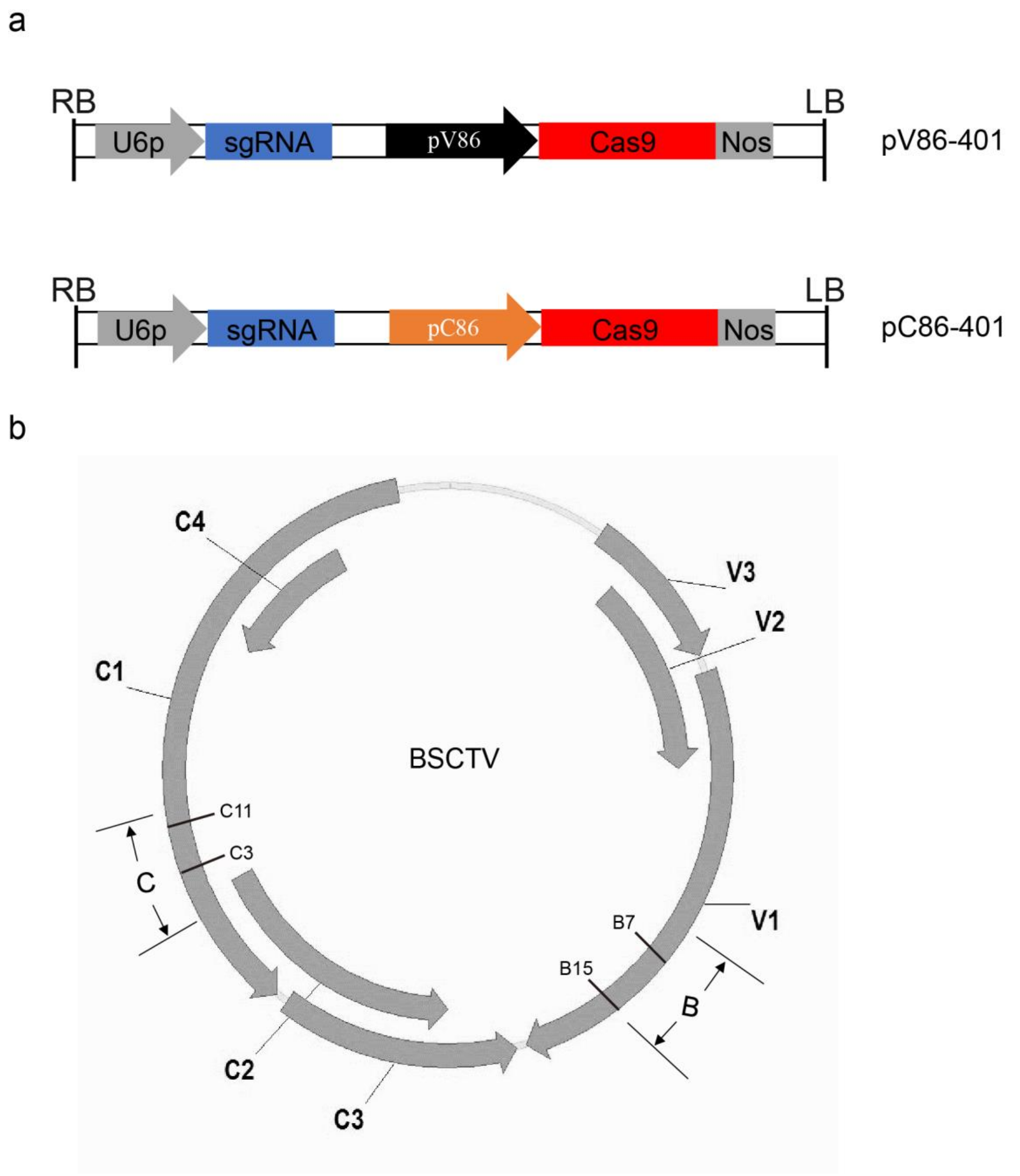

Figure S3. Constructs for the two BSCTV-inducible systems. The sgRNA is driven by the AtU6 promoter and Cas9 by the pV86 (black) or pC86 promoter (orange) (a). sgRNAs B7 and B15 are located in the V1 open reading frame and C3 and C11 in the C1 open reading frame (b).

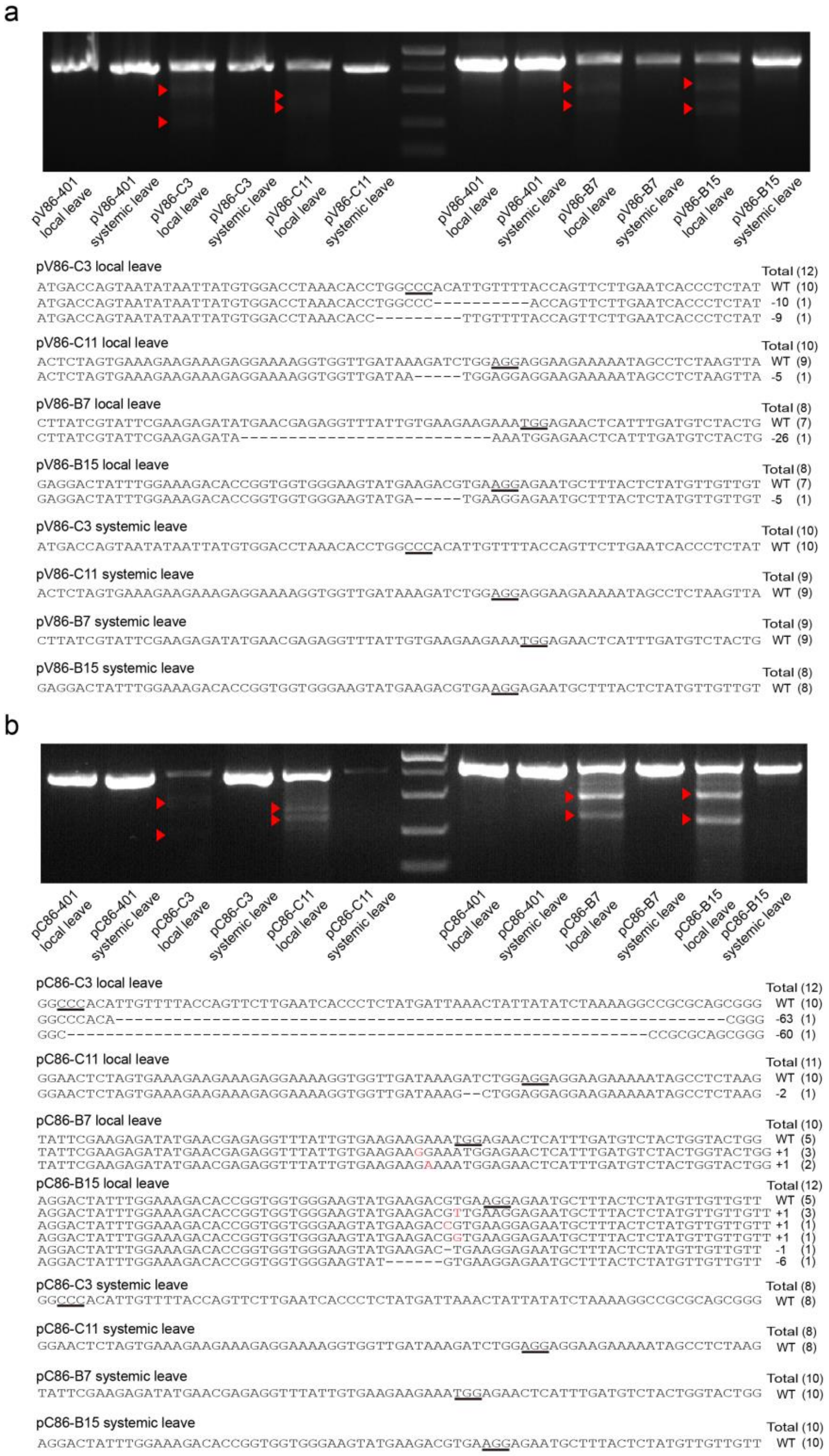

Figure S4. Mutations induced in the BSCTV genome by the inducible pV86-401 (a) and pC86-401 (b) constructs. Red arrowheads indicate fragments generated by T7E1. −/+ indicates deletions/insertions of nucleotides. The number to the right of each row indicate numbers of independent plasmid clones found after cloning and sequencing of PCR amplicons. For systemic leaves, a second round PCR was performed to amplify the rare virus.

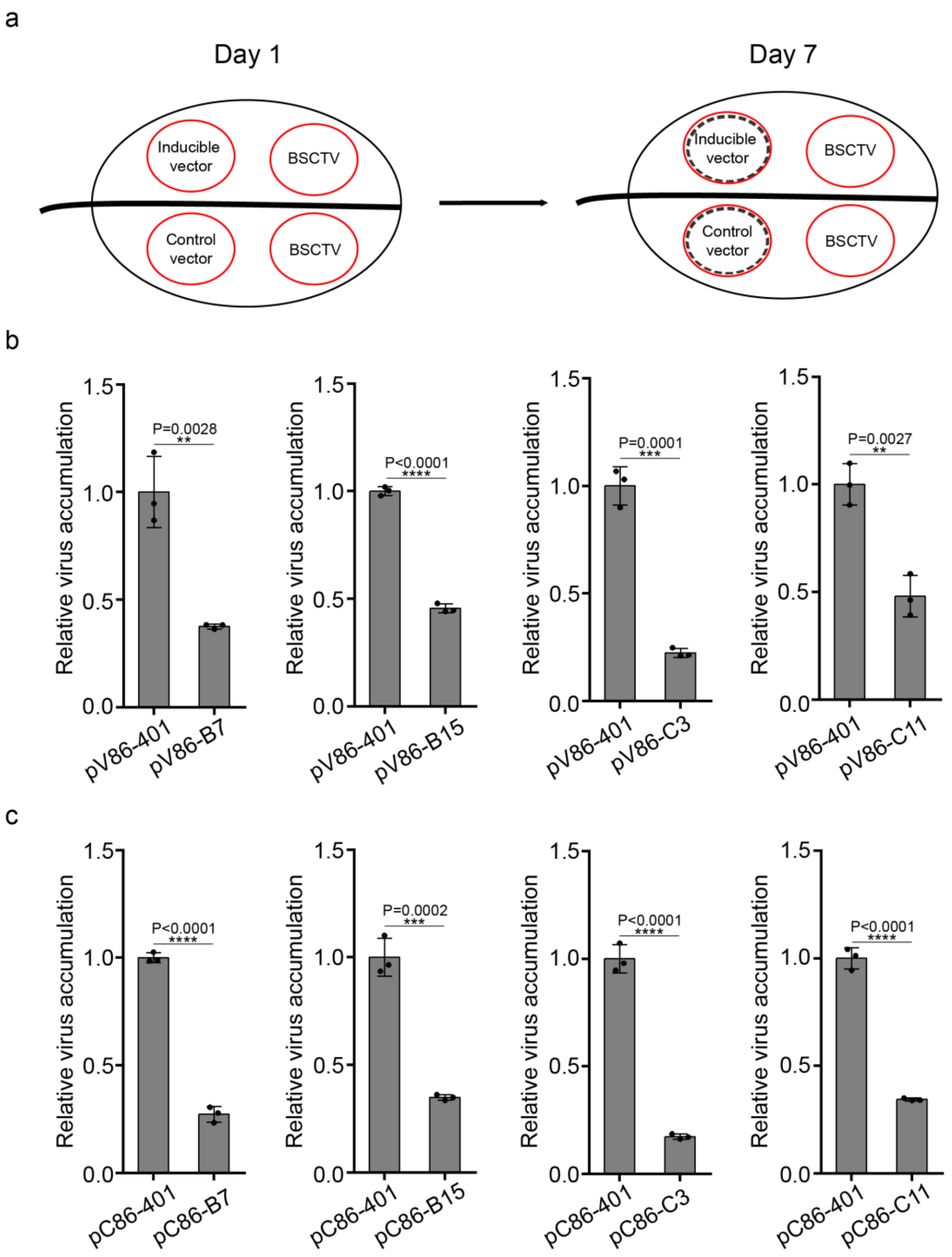

Figure S5. qPCR analysis of the sensitivity and efficiency of the inducible pV86-401 (b) and pC86-401 (c) combinations for inhibiting virus replication (n = 3) in *N. benthamiana* plants. (a) Strategy for testing whether the inducible CRISPR/Cas9 system reduced the amount of replicating virus. The DNA in the bottom regions (marked by dashed lines) was extracted and viral accumulation was measured by qPCR. All values represent means ± S.D. $^{**}P < 0.01$, $^{***}P < 0.001$, $^{****}P < 0.0001$; two-tailed Student's t-test.

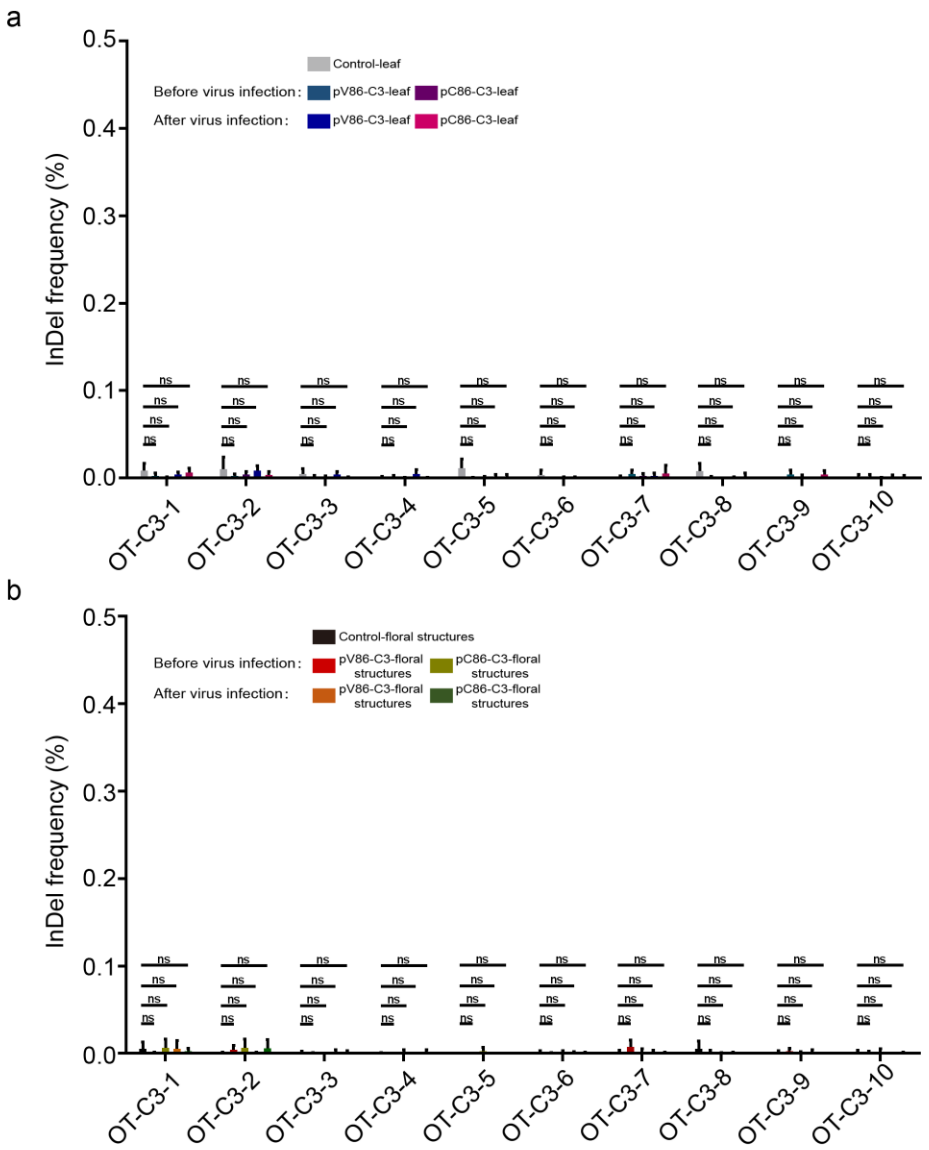

Figure S6. Indel frequencies at ten candidate sites in the leaves (a) and floral structures (b) of transgenic pV86-C3 and pC86-C3 *Arabidopsis* plants were similar to the frequencies in non-transgenic control *Arabidopsis* plants (n = 3). All values represent means ± S.D. ns, not significant by two-tailed Student's t-test.

Table S1. Potential off-target sites for C3 sgRNA in *Arabidopsis*.

sgRNA ID	Mismatches	Off-target sequence[a]	Chromosome	Position	Direction
C3	0	CCCACATTGTTTTACCAGTTCTT			
OT-C3-1	3	AAGtAaTGGTAAgACAATGTCGG	chr4	15495282	+
OT-C3-2	3	AAGAAaTGGTgAAACAgTGTAAG	chr1	2064439	-
OT-C3-3	3	AAGAACatGTAAAACAATGaAAG	chr3	7075914	+
OT-C3-4	4	AAGgAaaGaTAAAACAATGTCAG	chr5	20031389	+
OT-C3-5	4	AAGtAtTcGTcAAACAATGTTGG	chr3	17560887	+
OT-C3-6	4	tAGAAaTGGatAAACAATGTAAG	chr3	22066699	+
OT-C3-7	4	AAGAgCTcGTAtAAaAATGTAGG	chr4	12148731	+
OT-C3-8	4	AAaAACTaGTgAAACcATGTGGG	chr3	4078517	-
OT-C3-9	4	AgGAACaGGaAAAACAATGgCGG	chr3	694395	+
OT-C3-10	4	AAGAAagGGaAAAACAATGgCGG	chr3	4712965	-

[a]The mismatch bases in the ten potential off-target sites are shown as lower case letters. The PAM motif is underlined.

Table S2. Indel frequencies detected by deep sequencing of transgenic C3 and control *Arabidopsis* plants.

Material for deep sequencing	Off-target site	Independent experiment repeat	InDel reads	Total reads	InDel frequency (%)	Primers and bar codes[a]
C3	OT-C3-1	R1	4	16105	0.0248	F: GACTAGTATTGCTTTATGTGTGTCTTGGTTTT<br>R: CAATCTGAACCGTCCAAACTTC
		R2	13	71363	0.0182	F: GTTTCGTTGCTTTATGTGTGTCTTGGTTTT<br>R: CAATCTGAACCGTCCAAACTTC
		R3	11	67640	0.0163	F: AACGTGATTTGCTTTATGTGTGTCTTGGTTTT<br>R: CAATCTGAACCGTCCAAACTTC
	OT-C3-2	R1	138	199216	0.0693	F: TTAGGCATAGATACTCATACCGCTTC<br>R: GTTAGTGAATGTGAATTTGCTTTT
		R2	91	67036	0.1357	F: CGTACGATAGATACTCATACCGCTTC<br>R: GTTAGTGAATGTGAATTTGCTTTT
		R3	78	93577	0.0834	F: AAACATCGATAGATACTCATACCGCTTC<br>R: GTTAGTGAATGTGAATTTGCTTTT
	OT-C3-3	R1	157	785808	0.02	F: ACAGTGCTCCATATACCCTCATTAGC<br>R: GTTGTTCTGAATAAGGCTGACCAAG
		R2	19	53449	0.0355	F: ACTATGCACTCCATATACCCTCATTAGC<br>R: GTTGTTCTGAATAAGGCTGACCAAG
		R3	10	54059	0.0185	F: GAGTGGCTCCATATACCCTCATTAGC<br>R: GTTGTTCTGAATAAGGCTGACCAAG
	OT-C3-4	R1	32	507149	0.0063	F: CAGATCGAGTAGATGGCAGGAGAAAA<br>R: CTCATAAGTCCAATGCCCAG
		R2	4	67557	0.0059	F: GGTAGCGAGTAGATGGCAGGAGAAAA<br>R: CTCATAAGTCCAATGCCCAG
		R3	9	83131	0.0108	F: AGAGTCAAGAGTAGATGGCAGGAGAAAA<br>R: CTCATAAGTCCAATGCCCAG
	OT-C3-5	R1	47	317358	0.0148	F: GATCAGTTCTTCTGATAGTGTCACTG<br>R: CACTTAAAAAAACGTAATGTTAC
		R2	8	39011	0.0205	F: ACCACTGTTTCTTCTGATAGTGTCACTG<br>R: CACTTAAAAAAACGTAATGTTAC
		R3	9	49055	0.0183	F: AGATCGCATTCTTCTGATAGTGTCACTG<br>R: CACTTAAAAAAACGTAATGTTAC
	OT-C3-6	R1	136	468604	0.029	F: GGCTACGCTTCTCTTATCTTTTCTAC<br>R: CTTTTTTTTTTCTGTAGACC
		R2	11	52655	0.0209	F: ATGAGCGCTTCTCTTATCTTTTCTAC<br>R: CTTTTTTTTTTCTGTAGACC
		R3	5	44668	0.0112	F: ACATTGGCGCTTCTCTTATCTTTTCTAC<br>R: CTTTTTTTTTTCTGTAGACC

	OT-C3-7	R1	861	454453	0.1895	F: AGTCAAGTGTAGTGGAAGAGAGCTGG<br>R: ATGTATGTGTAAATTGTGAG
		R2	136	81295	0.1673	F: ATTCCTGTGTAGTGGAAGAGAGCTGG<br>R: ATGTATGTGTAAATTGTGAG
		R3	125	62347	0.2005	F: CAGATCTGGTGTAGTGGAAGAGAGCTGG<br>R: ATGTATGTGTAAATTGTGAG
	OT-C3-8	R1	4089	343576	1.1901	F: ATGTCAATATAATACTGTATTGGGCTTGG<br>R: GTTACCTTTAGCTTCTCTAG
		R2	426	114944	0.3706	F: CCGTGAGAATATAATACTGTATTGGGCTTGG<br>R: GTTACCTTTAGCTTCTCTAG
		R3	102	15547	0.6561	F: GAGCTGAAATATAATACTGTATTGGGCTTGG<br>R: GTTACCTTTAGCTTCTCTAG
	OT-C3-9	R1	3	22686	0.0132	F: CAACTATAGCTACCTCAACCGCCAAC<br>R: TCCATGGTCTATTTGGGTGTC
		R2	14	59188	0.0237	F: CAACTATAGCTACCTCAACCGCCAAC<br>R: TCCATGGTCTATTTGGGTGTC
		R3	5	34090	0.0147	F: GATAGACATAGCTACCTCAACCGCCAAC<br>R: TCCATGGTCTATTTGGGTGTC
	OT-C3-10	R1	3	21935	0.0137	F: CACCGGTGAAACCACTTCAAACTCTTCC<br>R: TTGTTTGACGACGACGTTTC
		R2	9	38525	0.0234	F: CACCGGTGAAACCACTTCAAACTCTTCC<br>R: TTGTTTGACGACGACGTTTC
		R3	7	51520	0.0136	F: ACAAGCTATGAAACCACTTCAAACTCTTCC<br>R: TTGTTTGACGACGACGTTTC
WT	OT-C3-1	R1	0	49387	0	F: ATCACGTTGCTTTATGTGTGTCTTGGTTTT<br>R: CAATCTGAACCGTCCAAACTTC
		R2	1	189825	0.0005	F: ATCACGTTGCTTTATGTGTGTCTTGGTTTT<br>R: CAATCTGAACCGTCCAAACTTC
		R3	10	252449	0.004	F: GTTTCGTTGCTTTATGTGTGTCTTGGTTTT<br>R: CAATCTGAACCGTCCAAACTTC
	OT-C3-2	R1	12	81718	0.0147	F: CGATGTATAGATACTCATACCGCTTC<br>R: GTTAGTGAATGTGAATTTGCTTTT
		R2	5	247317	0.002	F: CGATGTATAGATACTCATACCGCTTC<br>R: GTTAGTGAATGTGAATTTGCTTTT
		R3	5	236444	0.0021	F: CGTACGATAGATACTCATACCGCTTC<br>R: GTTAGTGAATGTGAATTTGCTTTT
	OT-C3-3	R1	0	39082	0	F: TTAGGCCTCCATATACCCTCATTAGC<br>R: GTTGTTCTGAATAAGGCTGACCAAG
		R2	0	55577	0	F: TTAGGCCTCCATATACCCTCATTAGC<br>R: GTTGTTCTGAATAAGGCTGACCAAG
		R3	0	104611	0	F: GAGTGGCTCCATATACCCTCATTAGC<br>R: GTTGTTCTGAATAAGGCTGACCAAG
	OT-C3-4	R1	3	55938	0.0054	F: TGACCAGAGTAGATGGCAGGAGAAAA<br>R: CTCATAAGTCCAATGCCCAG

		R2	0	81201	0	F: TGACCAGAGTAGATGGCAGGAGAAAA<br>R: CTCATAAGTCCAATGCCCAG
		R3	0	194981	0	F: GGTAGCGAGTAGATGGCAGGAGAAAA<br>R: CTCATAAGTCCAATGCCCAG
	OT-C3-5	R1	0	36204	0	F: ACAGTGTTCTTCTGATAGTGTCACTG<br>R: CACTTAAAAAAACGTAATGTTAC
		R2	0	112484	0	F: ACAGTGTTCTTCTGATAGTGTCACTG<br>R: CACTTAAAAAAACGTAATGTTAC
		R3	0	113921	0	F: ACTGATTTCTTCTGATAGTGTCACTG<br>R: CACTTAAAAAAACGTAATGTTAC
	OT-C3-6	R1	0	18565	0	F: TAATCGGCTTCTCTTATCTTTTCTAC<br>R: CTTTTTTTTTTCTGTAGACC
		R2	9	64017	0.0141	F: TAATCGGCTTCTCTTATCTTTTCTAC<br>R: CTTTTTTTTTTCTGTAGACC
		R3	0	1654	0	F: ATGAGCGCTTCTCTTATCTTTTCTAC<br>R: CTTTTTTTTTTCTGTAGACC
	OT-C3-7	R1	0	137351	0	F: CAGATCGTGTAGTGGAAGAGAGCTGG<br>R: ATGTATGTGTAAATTGTGAG
		R2	18	218972	0.0082	F: CAGATCGTGTAGTGGAAGAGAGCTGG<br>R: ATGTATGTGTAAATTGTGAG
		R3	24	302896	0.0079	F: ATTCCTGTGTAGTGGAAGAGAGCTGG<br>R: ATGTATGTGTAAATTGTGAG
	OT-C3-8	R1	0	155851	0	F: GAGCTGAAATATAATACTGTATTGGGCTTGG<br>R: GTTACCTTTAGCTTCTCTAG
		R2	17	204649	0.0083	F: ACTTGAATATAATACTGTATTGGGCTTGG<br>R: GTTACCTTTAGCTTCTCTAG
		R3	19	143249	0.0133	F: CAAAAGATATAATACTGTATTGGGCTTGG<br>R: GTTACCTTTAGCTTCTCTAG
	OT-C3-9	R1	2	24927	0.008	F: GATCAGTAGCTACCTCAACCGCCAAC<br>R: TCCATGGTCTATTTGGGTGTC
		R2	3	93319	0.0032	F: GATCAGTAGCTACCTCAACCGCCAAC<br>R: TCCATGGTCTATTTGGGTGTC
		R3	1	65265	0.0015	F: CAACTATAGCTACCTCAACCGCCAAC<br>R: TCCATGGTCTATTTGGGTGTC
	OT-C3-10	R1	1	26248	0.0038	F: TACAGCTGAAACCACTTCAAACTCTTCC<br>R: TTGTTTGACGACGACGTTTC
		R2	0	38128	0	F: TACAGCTGAAACCACTTCAAACTCTTCC<br>R: TTGTTTGACGACGACGTTTC
		R3	0	88703	0	F: CACCGGTGAAACCACTTCAAACTCTTCC<br>R: TTGTTTGACGACGACGTTTC

[a]The barcodes used for deep sequencing are marked in red.

Table S3. Relative levels of virus accumulation for each of the four target sites in the inducible systems.

Material used in qPCR analysis	ID	Relative virus accumulation in each experimental repeat		
		1	2	3
Local leaves	pV86-401	1.1942	0.9556	0.8503
	pV86-C3	0.2635	0.0276	0.0722
	pV86-C11	0.4098	0.2551	0.5181
	pV86-B7	0.0399	0.2417	0.0533
	pV86-B15	0.0439	0.1488	0.2343
Systemic leaves	pV86-401	0.9932	0.9125	1.0943
	pV86-C3	0.0013	0.0045	0.0017
	pV86-C11	0.1476	0.2707	0.2003
	pV86-B7	0.0001	0.0002	0.0002
	pV86-B15	9E-05	0.0001	0.0007
Local leaves	pC86-401	1.1052	0.8941	1.0007
	pC86-C3	0.0149	0.0307	0.0348
	pC86-C11	0.0049	0.0012	0.0071
	pC86-B7	0.1846	0.0191	0.104
	pC86-B15	0.0452	0.0997	0.1587
Systemic leaves	pC86-401	0.9089	1.0631	1.0281
	pC86-C3	0.0356	0.0015	0.0003
	pC86-C11	0.0078	0.0182	0.0023
	pC86-B7	0.0147	0.0073	0.0005
	pC86-B15	0.0048	0.0157	0.0178

Table S4. The relative viral load detected from the experiment of inhibiting replicating virus.

Group	ID	Relative virus accumulation from each experiment repeat		
		1	2	3
1	pV86-401	0.8996	1.0691	1.0312
	pV86-C3	0.2098	0.2142	0.2483
2	pV86-401	0.9043	0.9977	1.098
	pV86-C11	0.4632	0.3923	0.5839
3	pV86-401	1.185	0.946	0.8687
	pV86-B7	0.382	0.3627	0.3836
4	pV86-401	1.0192	1.0026	0.9781
	pV86-B15	0.4407	0.446	0.479
5	pC86-401	0.947	0.9793	1.0733
	pC86-C3	0.162	0.1711	0.1856
6	pC86-401	0.9448	1.0132	1.042
	pC86-C11	0.3511	0.3389	0.342
7	pC86-401	0.9851	0.9896	1.0253
	pC86-B7	0.3048	0.2786	0.2329
8	pC86-401	0.9654	0.934	1.1006
	pC86-B15	0.3352	0.3484	0.3606

Table S5. InDel frequencies detected by deep sequencing with their primers in transgenic pV86-C3, pC86-C3 and control *Arabidopsis* plants.

	Material for deep sequencing	Off-target site	Independent experiment repeat	InDel reads	Total reads	InDel frequency	Primers and barcodes[a]
Before virus infection	pV86-C3 leaf	OT-C3-1	R1	5	821295	6.09E-06	F: GTTTCGTTGCTTTATGTGTGTCTTGGTTTT<br>R: CAATCTGAACCGTCCAAACTTC
			R2	0	195521	0	F: GTTTCGTTGCTTTATGTGTGTCTTGGTTTT<br>R: CAATCTGAACCGTCCAAACTTC
			R3	5	86218	5.8E-05	F: ACGCTCGATTGCTTTATGTGTGTCTTGGTTTT<br>R: CAATCTGAACCGTCCAAACTTC
		OT-C3-2	R1	43	871531	4.93E-05	F: CGTACGATAGATACTCATACCGCTTC<br>R: GTTAGTGAATGTGAATTTGCTTTT
			R2	1	215289	4.64E-06	F: CGTACGATAGATACTCATACCGCTTC<br>R: GTTAGTGAATGTGAATTTGCTTTT
			R3	1	100114	9.99E-06	F: ACGTATCAATAGATACTCATACCGCTTC<br>R: GTTAGTGAATGTGAATTTGCTTTT
		OT-C3-3	R1	1	214992	4.65E-06	F: GAGTGGCTCCATATACCCTCATTAGC<br>R: GTTGTTCTGAATAAGGCTGACCAAG
			R2	3	101658	2.95E-05	F: GAGTGGCTCCATATACCCTCATTAGC<br>R: GTTGTTCTGAATAAGGCTGACCAAG
			R3	1	165140	6.06E-06	F: ACTATGCACTCCATATACCCTCATTAGC<br>R: GTTGTTCTGAATAAGGCTGACCAAG
		OT-C3-4	R1	0	627550	0	F: GGTAGCGAGTAGATGGCAGGAGAAAA<br>R: CTCATAAGTCCAATGCCCAG
			R2	4	143803	2.78E-05	F: GGTAGCGAGTAGATGGCAGGAGAAAA<br>R: CTCATAAGTCCAATGCCCAG
			R3	0	132898	0	F: AGAGTCAAGAGTAGATGGCAGGAGAAAA<br>R: CTCATAAGTCCAATGCCCAG
		OT-C3-5	R1	1	252144	3.97E-06	F: ACTGATTTCTTCTGATAGTGTCACTG<br>R: CACTTAAAAAAACGTAATGTTAC
			R2	0	90957	0	F: ACTGATTTCTTCTGATAGTGTCACTG<br>R: CACTTAAAAAAACGTAATGTTAC
			R3	0	33649	0	F: AGATCGCATTCTTCTGATAGTGTCACTG<br>R: CACTTAAAAAAACGTAATGTTAC
		OT-C3-6	R1	0	175567	0	F: ATGAGCGCTTCTCTTATCTTTTCTAC<br>R: CTTTTTTTTTTCTGTAGACC
			R2	0	34403	0	F: ATGAGCGCTTCTCTTATCTTTTCTAC<br>R: CTTTTTTTTTTCTGTAGACC
			R3	0	117065	0	F: AGCAGGAAGCTTCTCTTATCTTTTCTAC<br>R: CTTTTTTTTTTCTGTAGACC
		OT-C3-	R1	40	936141	4.27E-05	F: ATTCCTGTGTAGTGGAAGAGAGCTGG

		7					R: ATGTATGTGTAAATTGTGAG
			R2	20	219013	9.13E-05	F: ATTCCTGTGTAGTGGAAGAGAGCTGG<br>R: ATGTATGTGTAAATTGTGAG
			R3	0	145886	0	F: AGTCACTAGTGTAGTGGAAGAGAGCTGG<br>R: ATGTATGTGTAAATTGTGAG
		OT-C3-8	R1	0	247472	0	F: CAAAAGATATAATACTGTATTGGGCTTGG<br>R: GTTACCTTTAGCTTCTCTAG
			R2	2	112807	1.77E-05	F: CAAAAGATATAATACTGTATTGGGCTTGG<br>R: GTTACCTTTAGCTTCTCTAG
			R3	0	33343	0	F: ATCCTGTAATATAATACTGTATTGGGCTTGG<br>R: GTTACCTTTAGCTTCTCTAG
		OT-C3-9	R1	2	134527	1.49E-05	F: CAACTATAGCTACCTCAACCGCCAAC<br>R: TCCATGGTCTATTTGGGTGTC
			R2	0	30261	0	F: CAACTATAGCTACCTCAACCGCCAAC<br>R: TCCATGGTCTATTTGGGTGTC
			R3	4	40807	9.8E-05	F: ATTGAGGATAGCTACCTCAACCGCCAAC<br>R: TCCATGGTCTATTTGGGTGTC
		OT-C3-10	R1	3	75484	3.97E-05	F: CACCGGTGAAACCACTTCAAACTCTTCC<br>R: TTGTTTGACGACGACGTTTC
			R2	1	118095	8.47E-06	F: CACCGGTGAAACCACTTCAAACTCTTCC<br>R: TTGTTTGACGACGACGTTTC
			R3	0	107007	0	F: CAACCACATGAAACCACTTCAAACTCTTCC<br>R: TTGTTTGACGACGACGTTTC
	pV86-C3 floral structures	OT-C3-1	R1	1	88779	1.13E-05	F: ATCACGTTGCTTTATGTGTGTCTTGGTTTT<br>R: CAATCTGAACCGTCCAAACTTC
			R2	1	153539	6.51E-06	F: ATCACGTTGCTTTATGTGTGTCTTGGTTTT<br>R: CAATCTGAACCGTCCAAACTTC
			R3	0	111371	0	F: AACGTGATTTGCTTTATGTGTGTCTTGGTTTT<br>R: CAATCTGAACCGTCCAAACTTC
		OT-C3-2	R1	45	1107572	4.06E-05	F: CGATGTATAGATACTCATACCGCTTC<br>R: GTTAGTGAATGTGAATTTGCTTTT
			R2	1	11077	9.03E-05	F: CGATGTATAGATACTCATACCGCTTC<br>R: GTTAGTGAATGTGAATTTGCTTTT
			R3	0	16914	0	F: AAACATCGATAGATACTCATACCGCTTC<br>R: GTTAGTGAATGTGAATTTGCTTTT
		OT-C3-3	R1	1	119537	8.37E-06	F: TTAGGCCTCCATATACCCTCATTAGC<br>R: GTTGTTCTGAATAAGGCTGACCAAG
			R2	0	13802	0	F: TTAGGCCTCCATATACCCTCATTAGC<br>R: GTTGTTCTGAATAAGGCTGACCAAG
			R3	10	135515	7.38E-05	F: ATGCCTAACTCCATATACCCTCATTAGC<br>R: GTTGTTCTGAATAAGGCTGACCAAG
		OT-C3-4	R1	1	374103	2.67E-06	F: TGACCAGAGTAGATGGCAGGAGAAAA<br>R: CTCATAAGTCCAATGCCCAG

			R2	0	154429	0	F: TGACCAGAGTAGATGGCAGGAGAAAA<br>R: CTCATAAGTCCAATGCCCAG
			R3	0	160936	0	F: AGTGGTCAGAGTAGATGGCAGGAGAAAA<br>R: CTCATAAGTCCAATGCCCAG
		OT-C3-5	R1	0	233549	0	F: ACAGTGTTCTTCTGATAGTGTCACTG<br>R: CACTTAAAAAAACGTAATGTTAC
			R2	0	82100	0	F: ACAGTGTTCTTCTGATAGTGTCACTG<br>R: CACTTAAAAAAACGTAATGTTAC
			R3	0	21259	0	F: ACCACTGTTTCTTCTGATAGTGTCACTG<br>R: CACTTAAAAAAACGTAATGTTAC
		OT-C3-6	R1	2	321599	6.22E-06	F: TAATCGGCTTCTCTTATCTTTTCTAC<br>R: CTTTTTTTTTTCTGTAGACC
			R2	0	41659	0	F: TAATCGGCTTCTCTTATCTTTTCTAC<br>R: CTTTTTTTTTTCTGTAGACC
			R3	3	87336	3.44E-05	F: ACATTGGCGCTTCTCTTATCTTTTCTAC<br>R: CTTTTTTTTTTCTGTAGACC
		OT-C3-7	R1	38	655325	5.8E-05	F: CAGATCGTGTAGTGGAAGAGAGCTGG<br>R: ATGTATGTGTAAATTGTGAG
			R2	2	12494	0.00016	F: CAGATCGTGTAGTGGAAGAGAGCTGG<br>R: ATGTATGTGTAAATTGTGAG
			R3	0	129345	0	F: CAGATCTGGTGTAGTGGAAGAGAGCTGG<br>R: ATGTATGTGTAAATTGTGAG
		OT-C3-8	R1	0	428306	0	F: ACTTGAATATAATACTGTATTGGGCTTGG<br>R: GTTACCTTTAGCTTCTCTAG
			R2	2	48158	4.15E-05	F: ACTTGAATATAATACTGTATTGGGCTTGG<br>R: GTTACCTTTAGCTTCTCTAG
			R3	0	53203	0	F: CATCAAGTATATAATACTGTATTGGGCTTGG<br>R: GTTACCTTTAGCTTCTCTAG
		OT-C3-9	R1	1	130109	7.69E-06	F: GATCAGTAGCTACCTCAACCGCCAAC<br>R: TCCATGGTCTATTTGGGTGTC
			R2	1	16324	6.13E-05	F: GATCAGTAGCTACCTCAACCGCCAAC<br>R: TCCATGGTCTATTTGGGTGTC
			R3	1	281781	3.55E-06	F: CGCTGATCTAGCTACCTCAACCGCCAAC<br>R: TCCATGGTCTATTTGGGTGTC
		OT-C3-10	R1	1	32969	3.03E-05	F: TACAGCTGAAACCACTTCAAACTCTTCC<br>R: TTGTTTGACGACGACGTTTC
			R2	0	90191	0	F: TACAGCTGAAACCACTTCAAACTCTTCC<br>R: TTGTTTGACGACGACGTTTC
			R3	5	269732	1.85E-05	F: ACAAGCTATGAAACCACTTCAAACTCTTCC<br>R: TTGTTTGACGACGACGTTTC
	pC86-C3 leaf	OT-C3-1	R1	0	12571	0	F: GACTAGTATTGCTTTATGTGTGTCTTGGTTTT<br>R: CAATCTGAACCGTCCAAACTTC
			R2	1	104907	9.53E-06	F: GTTTCGTTGCTTTATGTGTGTCTTGGTTTT<br>R: CAATCTGAACCGTCCAAACTTC

			R3	0	26463	0	F: ACGCTCGATTGCTTTATGTGTGTCTTGGTTTT<br>R: CAATCTGAACCGTCCAAACTTC
		OT-C3-2	R1	20	1470627	1.36E-05	F: CGTACGATAGATACTCATACCGCTTC<br>R: GTTAGTGAATGTGAATTTGCTTTT
			R2	10	136743	7.31E-05	F: CGTACGATAGATACTCATACCGCTTC<br>R: GTTAGTGAATGTGAATTTGCTTTT
			R3	1	26565	3.76E-05	F: ACGTATCAATAGATACTCATACCGCTTC<br>R: GTTAGTGAATGTGAATTTGCTTTT
		OT-C3-3	R1	1	132928	7.52E-06	F: GAGTGGCTCCATATACCCTCATTAGC<br>R: GTTGTTCTGAATAAGGCTGACCAAG
			R2	2	75945	2.63E-05	F: GAGTGGCTCCATATACCCTCATTAGC<br>R: GTTGTTCTGAATAAGGCTGACCAAG
			R3	0	71067	0	F: ACTATGCACTCCATATACCCTCATTAGC<br>R: GTTGTTCTGAATAAGGCTGACCAAG
		OT-C3-4	R1	2	438760	4.56E-06	F: GGTAGCGAGTAGATGGCAGGAGAAAA<br>R: CTCATAAGTCCAATGCCCAG
			R2	0	155860	0	F: GGTAGCGAGTAGATGGCAGGAGAAAA<br>R: CTCATAAGTCCAATGCCCAG
			R3	0	65204	0	F: AGAGTCAAGAGTAGATGGCAGGAGAAAA<br>R: CTCATAAGTCCAATGCCCAG
		OT-C3-5	R1	0	165144	0	F: ACTGATTTCTTCTGATAGTGTCACTG<br>R: CACTTAAAAAAACGTAATGTTAC
			R2	2	121345	1.65E-05	F: ACTGATTTCTTCTGATAGTGTCACTG<br>R: CACTTAAAAAAACGTAATGTTAC
			R3	0	82031	0	F: AGATCGCATTCTTCTGATAGTGTCACTG<br>R: CACTTAAAAAAACGTAATGTTAC
		OT-C3-6	R1	2	869497	2.3E-06	F: ATGAGCGCTTCTCTTATCTTTTCTAC<br>R: CTTTTTTTTTTCTGTAGACC
			R2	1	97175	1.03E-05	F: ATGAGCGCTTCTCTTATCTTTTCTAC<br>R: CTTTTTTTTTTCTGTAGACC
			R3	1	101866	9.82E-06	F: AGCAGGAAGCTTCTCTTATCTTTTCTAC<br>R: CTTTTTTTTTTCTGTAGACC
		OT-C3-7	R1	56	1021602	5.48E-05	F: ATTCCTGTGTAGTGGAAGAGAGCTGG<br>R: ATGTATGTGTAAATTGTGAG
			R2	0	86113	0	F: ATTCCTGTGTAGTGGAAGAGAGCTGG<br>R: ATGTATGTGTAAATTGTGAG
			R3	1	52823	1.89E-05	F: AGTCACTAGTGTAGTGGAAGAGAGCTGG<br>R: ATGTATGTGTAAATTGTGAG
		OT-C3-8	R1	0	173719	0	F: CAAAAGATATAATACTGTATTGGGCTTGG<br>R: GTTACCTTTAGCTTCTCTAG
			R2	0	5858	0	F: CAAAAGATATAATACTGTATTGGGCTTGG<br>R: GTTACCTTTAGCTTCTCTAG
			R3	0	19138	0	F: ATCCTGTAATATAATACTGTATTGGGCTTGG<br>R: GTTACCTTTAGCTTCTCTAG

		OT-C3-9	R1	2	54886	3.64E-05	F: CAACTATAGCTACCTCAACCGCCAAC<br>R: TCCATGGTCTATTTGGGTGTC
			R2	0	20697	0	F: CAACTATAGCTACCTCAACCGCCAAC<br>R: TCCATGGTCTATTTGGGTGTC
			R3	3	228724	1.31E-05	F: AACTCACCTAGCTACCTCAACCGCCAAC<br>R: TCCATGGTCTATTTGGGTGTC
		OT-C3-10	R1	0	34189	0	F: CACCGGTGAAACCACTTCAAACTCTTCC<br>R: TTGTTTGACGACGACGTTTC
			R2	0	35319	0	F: CACCGGTGAAACCACTTCAAACTCTTCC<br>R: TTGTTTGACGACGACGTTTC
			R3	2	296920	6.74E-06	F: CAACCACATGAAACCACTTCAAACTCTTCC<br>R: TTGTTTGACGACGACGTTTC
	pC86-C3 floral structures	OT-C3-1	R1	3	16966	0.00017	F: AACGTGATTTGCTTTATGTGTGTCTTGGTTTT<br>R: CAATCTGAACCGTCCAAACTTC
			R2	2	125692	1.59E-05	F: ATCACGTTGCTTTATGTGTGTCTTGGTTTT<br>R: CAATCTGAACCGTCCAAACTTC
			R3	0	80020	0	F: AACGTGATTTGCTTTATGTGTGTCTTGGTTTT<br>R: CAATCTGAACCGTCCAAACTTC
		OT-C3-2	R1	3	259474	1.16E-05	F: CGATGTATAGATACTCATACCGCTTC<br>R: GTTAGTGAATGTGAATTTGCTTTT
			R2	22	122748	0.00018	F: CGATGTATAGATACTCATACCGCTTC<br>R: GTTAGTGAATGTGAATTTGCTTTT
			R3	0	76531	0	F: AAACATCGATAGATACTCATACCGCTTC<br>R: GTTAGTGAATGTGAATTTGCTTTT
		OT-C3-3	R1	0	93771	0	F: TTAGGCCTCCATATACCCTCATTAGC<br>R: GTTGTTCTGAATAAGGCTGACCAAG
			R2	0	39079	0	F: TTAGGCCTCCATATACCCTCATTAGC<br>R: GTTGTTCTGAATAAGGCTGACCAAG
			R3	1	103622	9.65E-06	F: ATGCCTAACTCCATATACCCTCATTAGC<br>R: GTTGTTCTGAATAAGGCTGACCAAG
		OT-C3-4	R1	1	361689	2.76E-06	F: TGACCAGAGTAGATGGCAGGAGAAAA<br>R: CTCATAAGTCCAATGCCCAG
			R2	5	112351	4.45E-05	F: TGACCAGAGTAGATGGCAGGAGAAAA<br>R: CTCATAAGTCCAATGCCCAG
			R3	1	102784	9.73E-06	F: AGTGGTCA GAGTAGATGGCAGGAGAAAA<br>R: CTCATAAGTCCAATGCCCAG
		OT-C3-5	R1	0	55107	0	F: ACAGTGTTCTTCTGATAGTGTCACTG<br>R: CACTTAAAAAAACGTAATGTTAC
			R2	2	25991	7.69E-05	F: ACAGTGTTCTTCTGATAGTGTCACTG<br>R: CACTTAAAAAAACGTAATGTTAC
			R3	0	11946	0	F: ACCACTGTTTCTTCTGATAGTGTCACTG<br>R: CACTTAAAAAAACGTAATGTTAC
		OT-C3-6	R1	0	62128	0	F: TAATCGGCTTCTCTTATCTTTTCTAC<br>R: CTTTTTTTTTTCTGTAGACC

			R2	1	27875	3.59E-05	F: TAATCGGCTTCTCTTATCTTTTCTAC<br>R: CTTTTTTTTTTCTGTAGACC
			R3	2	111170	1.8E-05	F: ACATTGGCGCTTCTCTTATCTTTTCTAC<br>R: CTTTTTTTTTTCTGTAGACC
		OT-C3-7	R1	31	562000	5.52E-05	F: CAGATCGTGTAGTGGAAGAGAGCTGG<br>R: ATGTATGTGTAAATTGTGAG
			R2	1	176916	5.65E-06	F: CAGATCGTGTAGTGGAAGAGAGCTGG<br>R: ATGTATGTGTAAATTGTGAG
			R3	1	76410	1.31E-05	F: CAGATCTGGTGTAGTGGAAGAGAGCTGG<br>R: ATGTATGTGTAAATTGTGAG
		OT-C3-8	R1	1	342936	2.92E-06	F: ACTTGAATATAATACTGTATTGGGCTTGG<br>R: GTTACCTTTAGCTTCTCTAG
			R2	1	92118	1.09E-05	F: ACTTGAATATAATACTGTATTGGGCTTGG<br>R: GTTACCTTTAGCTTCTCTAG
			R3	0	14696	0	F: CATCAAGTATATAATACTGTATTGGGCTTGG<br>R: GTTACCTTTAGCTTCTCTAG
		OT-C3-9	R1	2	103172	1.94E-05	F: GATCAGTAGCTACCTCAACCGCCAAC<br>R: TCCATGGTCTATTTGGGTGTC
			R2	0	50235	0	F: GATCAGTAGCTACCTCAACCGCCAAC<br>R: TCCATGGTCTATTTGGGTGTC
			R3	1	65866	1.52E-05	F: CGCTGATCTAGCTACCTCAACCGCCAAC<br>R: TCCATGGTCTATTTGGGTGTC
		OT-C3-10	R1	1	16993	5.88E-05	F: TACAGCTGAAACCACTTCAAACTCTTCC<br>R: TTGTTTGACGACGACGTTTC
			R2	0	52218	0	F: TACAGCTGAAACCACTTCAAACTCTTCC<br>R: TTGTTTGACGACGACGTTTC
			R3	0	52840	0	F: ACAAGCTATGAAACCACTTCAAACTCTTCC<br>R: TTGTTTGACGACGACGTTTC
After virus infection	pV86-C3 leaf	OT-C3-1	R1	1	264290	3.78E-06	F: GTTTCGTTGCTTTATGTGTGTCTTGGTTTT<br>R: CAATCTGAACCGTCCAAACTTC
			R2	4	67680	5.91E-05	F: CGACTGGATTGCTTTATGTGTGTCTTGGTTTT<br>R: CAATCTGAACCGTCCAAACTTC
			R3	3	66400	4.52E-05	F: TATCAGCATTGCTTTATGTGTGTCTTGGTTTT<br>R: CAATCTGAACCGTCCAAACTTC
		OT-C3-2	R1	3	91649	3.27E-05	F: CGTACGATAGATACTCATACCGCTTC<br>R: GTTAGTGAATGTGAATTTGCTTTT
			R2	3	40189	7.46E-05	F: CGCATACAATAGATACTCATACCGCTTC<br>R: GTTAGTGAATGTGAATTTGCTTTT
			R3	4	28637	0.00014	F: CGTACGATAGATACTCATACCGCTTC<br>R: GTTAGTGAATGTGAATTTGCTTTT
		OT-C3-3	R1	0	100687	0	F: GAGTGGCTCCATATACCCTCATTAGC<br>R: GTTGTTCTGAATAAGGCTGACCAAG
			R2	3	71760	4.18E-05	F: CTCAATGACTCCATATACCCTCATTAGC<br>R: GTTGTTCTGAATAAGGCTGACCAAG

			R3	6	85626	7.01E-05	F: TCTTCACACTCCATATACCCTCATTAGC<br>R: GTTGTTCTGAATAAGGCTGACCAAG
		OT-C3-4	R1	1	128030	7.81E-06	F: GGTAGCGAGTAGATGGCAGGAGAAAA<br>R: CTCATAAGTCCAATGCCCAG
			R2	7	71201	9.83E-05	F: CTGAGCCAGAGTAGATGGCAGGAGAAAA<br>R: CTCATAAGTCCAATGCCCAG
			R3	2	59546	3.36E-05	F: TGAAGAGAGAGTAGATGGCAGGAGAAAA<br>R: CTCATAAGTCCAATGCCCAG
		OT-C3-5	R1	0	6697	0	F: ACTGATTTCTTCTGATAGTGTCACTG<br>R: CACTTAAAAAAACGTAATGTTAC
			R2	0	67905	0	F: CTGGCATATTCTTCTGATAGTGTCACTG<br>R: CACTTAAAAAAACGTAATGTTAC
			R3	2	46306	4.32E-05	F: TGGAACAATTCTTCTGATAGTGTCACTG<br>R: CACTTAAAAAAACGTAATGTTAC
		OT-C3-6	R1	1	103394	9.67E-06	F: ATGAGCGCTTCTCTTATCTTTTCTAC<br>R: CTTTTTTTTTTCTGTAGACC
			R2	0	63669	0	F: GAATCTGAGCTTCTCTTATCTTTTCTAC<br>R: CTTTTTTTTTTCTGTAGACC
			R3	0	69604	0	F: TGGCTTCAGCTTCTCTTATCTTTTCTAC<br>R: CTTTTTTTTTTCTGTAGACC
		OT-C3-7	R1	8	135723	5.89E-05	F: ATTCCTGTGTAGTGGAAGAGAGCTGG<br>R: ATGTATGTGTAAATTGTGAG
			R2	0	136431	0	F: CAAGACTAGTGTAGTGGAAGAGAGCTGG<br>R: ATGTATGTGTAAATTGTGAG
			R3	0	44781	0	F: TGGTGGTAGTGTAGTGGAAGAGAGCTGG<br>R: ATGTATGTGTAAATTGTGAG
		OT-C3-8	R1	0	32144	0	F: CAAAAGATATAATACTGTATTGGGCTTGG<br>R: GTTACCTTTAGCTTCTCTAG
			R2	1	94892	1.05E-05	F: GAGCTGAAATATAATACTGTATTGGGCTTGG<br>R: GTTACCTTTAGCTTCTCTAG
			R3	0	85475	0	F: TTCACGCAATATAATACTGTATTGGGCTTGG<br>R: GTTACCTTTAGCTTCTCTAG
		OT-C3-9	R1	0	22422	0	F: CAACTATAGCTACCTCAACCGCCAAC<br>R: TCCATGGTCTATTTGGGTGTC
			R2	0	51825	0	F: GATAGACATAGCTACCTCAACCGCCAAC<br>R: TCCATGGTCTATTTGGGTGTC
			R3	0	54777	0	F: AACTCACCTAGCTACCTCAACCGCCAAC<br>R: TCCATGGTCTATTTGGGTGTC
		OT-C3-10	R1	0	17407	0	F: CACCGGTGAAACCACTTCAAACTCTTCC<br>R: TTGTTTGACGACGACGTTTC
			R2	2	63133	3.17E-05	F: GCCACATATGAAACCACTTCAAACTCTTCC<br>R: TTGTTTGACGACGACGTTTC
			R3	1	74383	1.34E-05	F: AAGAGATCTGAAACCACTTCAAACTCTTCC<br>R: TTGTTTGACGACGACGTTTC

	pV86-C3 floral structures	OT-C3-1	R1	1	238214	4.2E-06	F: ATCACGTTGCTTTATGTGTGTCTTGGTTTT<br>R: CAATCTGAACCGTCCAAACTTC
			R2	10	61964	0.00016	F: GACTAGTATTGCTTTATGTGTGTCTTGGTTTT<br>R: CAATCTGAACCGTCCAAACTTC
			R3	4	65989	6.06E-05	F: GCGAGTAATTGCTTTATGTGTGTCTTGGTTTT<br>R: CAATCTGAACCGTCCAAACTTC
		OT-C3-2	R1	3	246290	1.22E-05	F: CGATGTATAGATACTCATACCGCTTC<br>R: GTTAGTGAATGTGAATTTGCTTTT
			R2	0	36317	0	F: CAATGGAAATAGATACTCATACCGCTTC<br>R: GTTAGTGAATGTGAATTTGCTTTT
			R3	2	74311	2.69E-05	F: GCTAACGAATAGATACTCATACCGCTTC<br>R: GTTAGTGAATGTGAATTTGCTTTT
		OT-C3-3	R1	0	65255	0	F: TTAGGCCTCCATATACCCTCATTAGC<br>R: GTTGTTCTGAATAAGGCTGACCAAG
			R2	4	90566	4.42E-05	F: CACTTCGACTCCATATACCCTCATTAGC<br>R: GTTGTTCTGAATAAGGCTGACCAAG
			R3	5	117086	4.27E-05	F: GCTCGGTACTCCATATACCCTCATTAGC<br>R: GTTGTTCTGAATAAGGCTGACCAAG
		OT-C3-4	R1	0	130602	0	F: TGACCAGAGTAGATGGCAGGAGAAAA<br>R: CTCATAAGTCCAATGCCCAG
			R2	0	69962	0	F: CAGCGTTAGAGTAGATGGCAGGAGAAAA<br>R: CTCATAAGTCCAATGCCCAG
			R3	1	119863	8.34E-06	F: GGAGAACAGAGTAGATGGCAGGAGAAAA<br>R: CTCATAAGTCCAATGCCCAG
		OT-C3-5	R1	0	108358	0	F: ACAGTGTTCTTCTGATAGTGTCACTG<br>R: CACTTAAAAAAACGTAATGTTAC
			R2	0	56972	0	F: CATACCAATTCTTCTGATAGTGTCACTG<br>R: CACTTAAAAAAACGTAATGTTAC
			R3	0	62863	0	F: GGTGCGAATTCTTCTGATAGTGTCACTG<br>R: CACTTAAAAAAACGTAATGTTAC
		OT-C3-6	R1	0	115349	0	F: TAATCGGCTTCTCTTATCTTTTCTAC<br>R: CTTTTTTTTTTCTGTAGACC
			R2	2	103209	1.94E-05	F: CCAGTTCAGCTTCTCTTATCTTTTCTAC<br>R: CTTTTTTTTTTCTGTAGACC
			R3	1	130208	7.68E-06	F: GTACGCAAGCTTCTCTTATCTTTTCTAC<br>R: CTTTTTTTTTTCTGTAGACC
		OT-C3-7	R1	8	198508	4.03E-05	F: CAGATCGTGTAGTGGAAGAGAGCTGG<br>R: ATGTATGTGTAAATTGTGAG
			R2	0	62510	0	F: CCGAAGTAGTGTAGTGGAAGAGAGCTGG<br>R: ATGTATGTGTAAATTGTGAG
			R3	4	25455	0.00016	F: GTCGTAGAGTGTAGTGGAAGAGAGCTGG<br>R: ATGTATGTGTAAATTGTGAG
		OT-C3-8	R1	2	123402	1.62E-05	F: ACTTGAATATAATACTGTATTGGGCTTGG<br>R: GTTACCTTTAGCTTCTCTAG

			R2	0	6768	0	F: CCGTGAGAATATAATACTGTATTGGGCTTGG<br>R: GTTACCTTTAGCTTCTCTAG
			R3	0	26279	0	F: GTCTGTCAATATAATACTGTATTGGGCTTGG<br>R: GTTACCTTTAGCTTCTCTAG
		OT-C3-9	R1	2	48476	4.13E-05	F: GATCAGTAGCTACCTCAACCGCCAAC<br>R: TCCATGGTCTATTTGGGTGTC
			R2	4	254278	1.57E-05	F: GATCAGTAGCTACCTCAACCGCCAAC<br>R: TCCATGGTCTATTTGGGTGTC
			R3	3	81444	3.68E-05	F: GTGTTCTATAGCTACCTCAACCGCCAAC<br>R: TCCATGGTCTATTTGGGTGTC
		OT-C3-10	R1	0	14703	0	F: TACAGCTGAAACCACTTCAAACTCTTCC<br>R: TTGTTTGACGACGACGTTTC
			R2	1	271767	3.68E-06	F: CGAACTTATGAAACCACTTCAAACTCTTCC<br>R: TTGTTTGACGACGACGTTTC
			R3	9	73245	0.00012	F: TAGGATGATGAAACCACTTCAAACTCTTCC<br>R: TTGTTTGACGACGACGTTTC
	pC86-C3 leaf	OT-C3-1	R1	1	166305	6.01E-06	F: GTTTCGTTGCTTTATGTGTGTCTTGGTTTT<br>R: CAATCTGAACCGTCCAAACTTC
			R2	10	105609	9.47E-05	F: CGACTGGATTGCTTTATGTGTGTCTTGGTTTT<br>R: CAATCTGAACCGTCCAAACTTC
			R3	4	46162	8.67E-05	F: TATCAGCATTGCTTTATGTGTGTCTTGGTTTT<br>R: CAATCTGAACCGTCCAAACTTC
		OT-C3-2	R1	3	91946	3.26E-05	F: CGTACGATAGATACTCATACCGCTTC<br>R: GTTAGTGAATGTGAATTTGCTTTT
			R2	8	106344	7.52E-05	F: CGCATACAATAGATACTCATACCGCTTC<br>R: GTTAGTGAATGTGAATTTGCTTTT
			R3	0	13321	0	F: GCTAACGAATAGATACTCATACCGCTTC<br>R: GTTAGTGAATGTGAATTTGCTTTT
		OT-C3-3	R1	1	106603	9.38E-06	F: GAGTGGCTCCATATACCCTCATTAGC<br>R: GTTGTTCTGAATAAGGCTGACCAAG
			R2	1	162675	6.15E-06	F: CTCAATGACTCCATATACCCTCATTAGC<br>R: GTTGTTCTGAATAAGGCTGACCAAG
			R3	0	53057	0	F: TCTTCACACTCCATATACCCTCATTAGC<br>R: GTTGTTCTGAATAAGGCTGACCAAG
		OT-C3-4	R1	1	147228	6.79E-06	F: GGTAGCGAGTAGATGGCAGGAGAAAA<br>R: CTCATAAGTCCAATGCCCAG
			R2	0	94656	0	F: CTGAGCCAGAGTAGATGGCAGGAGAAAA<br>R: CTCATAAGTCCAATGCCCAG
			R3	0	57359	0	F: TGAAGAGAGAGTAGATGGCAGGAGAAAA<br>R: CTCATAAGTCCAATGCCCAG
		OT-C3-5	R1	0	37232	0	F: ACTGATTTCTTCTGATAGTGTCACTG<br>R: CACTTAAAAAAACGTAATGTTAC
			R2	0	53098	0	F: CTGGCATATTCTTCTGATAGTGTCACTG<br>R: CACTTAAAAAAACGTAATGTTAC

			R3	2	46306	4.32E-05	F: TGGAACAATTCTTCTGATAGTGTCACTG<br>R: CACTTAAAAAAACGTAATGTTAC
		OT-C3-6	R1	0	104079	0	F: ATGAGCGCTTCTCTTATCTTTTCTAC<br>R: CTTTTTTTTTTCTGTAGACC
			R2	0	384679	0	F: ACATTGGCGCTTCTCTTATCTTTTCTAC<br>R: CTTTTTTTTTTCTGTAGACC
			R3	0	69604	0	F: TGGCTTCAGCTTCTCTTATCTTTTCTAC<br>R: CTTTTTTTTTTCTGTAGACC
		OT-C3-7	R1	35	224709	0.00016	F: ATTCCTGTGTAGTGGAAGAGAGCTGG<br>R: ATGTATGTGTAAATTGTGAG
			R2	0	95776	0	F: CAAGACTAGTGTAGTGGAAGAGAGCTGG<br>R: ATGTATGTGTAAATTGTGAG
			R3	0	6201	0	F: TGGTGGTAGTGTAGTGGAAGAGAGCTGG<br>R: ATGTATGTGTAAATTGTGAG
		OT-C3-8	R1	0	68204	0	F: CAAAAGATATAATACTGTATTGGGCTTGG<br>R: GTTACCTTTAGCTTCTCTAG
			R2	0	30943	0	F: GAGCTGAAATATAATACTGTATTGGGCTTGG<br>R: GTTACCTTTAGCTTCTCTAG
			R3	1	17235	5.8E-05	F: TTCACGCAATATAATACTGTATTGGGCTTGG<br>R: GTTACCTTTAGCTTCTCTAG
		OT-C3-9	R1	0	22642	0	F: CAACTATAGCTACCTCAACCGCCAAC<br>R: TCCATGGTCTATTTGGGTGTC
			R2	5	60134	8.31E-05	F: GATAGACATAGCTACCTCAACCGCCAAC<br>R: TCCATGGTCTATTTGGGTGTC
			R3	2	61165	3.27E-05	F: AACTCACCTAGCTACCTCAACCGCCAAC<br>R: TCCATGGTCTATTTGGGTGTC
		OT-C3-10	R1	0	11997	0	F: CACCGGTGAAACCACTTCAAACTCTTCC<br>R: TTGTTTGACGACGACGTTTC
			R2	0	46252	0	F: GCCACATATGAAACCACTTCAAACTCTTCC<br>R: TTGTTTGACGACGACGTTTC
			R3	1	31127	3.21E-05	F: AAGAGATCTGAAACCACTTCAAACTCTTCC<br>R: TTGTTTGACGACGACGTTTC
	pC86-C3 floral structures	OT-C3-1	R1	1	193662	5.16E-06	F: ATCACGTTGCTTTATGTGTGTCTTGGTTTT<br>R: CAATCTGAACCGTCCAAACTTC
			R2	6	97054	6.18E-05	F: GACTAGTATTGCTTTATGTGTGTCTTGGTTTT<br>R: CAATCTGAACCGTCCAAACTTC
			R3	0	41752	0	F: GCGAGTAATTGCTTTATGTGTGTCTTGGTTTT<br>R: CAATCTGAACCGTCCAAACTTC
		OT-C3-2	R1	3	246290	1.22E-05	F: CGATGTATAGATACTCATACCGCTTC<br>R: GTTAGTGAATGTGAATTTGCTTTT
			R2	18	106051	0.00017	F: CAATGGAAATAGATACTCATACCGCTTC<br>R: GTTAGTGAATGTGAATTTGCTTTT
			R3	20	74467	0.00027	F: GCTAACGAATAGATACTCATACCGCTTC<br>R: GTTAGTGAATGTGAATTTGCTTTT

		OT-C3-3	R1	1	50958	1.96E-05	F: TTAGGCCTCCATATACCCTCATTAGC<br>R: GTTGTTCTGAATAAGGCTGACCAAG
			R2	4	127493	3.14E-05	F: CACTTCGACTCCATATACCCTCATTAGC<br>R: GTTGTTCTGAATAAGGCTGACCAAG
			R3	13	78476	0.00017	F: GCTCGGTACTCCATATACCCTCATTAGC<br>R: GTTGTTCTGAATAAGGCTGACCAAG
		OT-C3-4	R1	5	107216	4.66E-05	F: TGACCAGAGTAGATGGCAGGAGAAAA<br>R: CTCATAAGTCCAATGCCCAG
			R2	0	101778	0	F: CAGCGTTAGAGTAGATGGCAGGAGAAAA<br>R: CTCATAAGTCCAATGCCCAG
			R3	0	80273	0	F: GGAGAACAGAGTAGATGGCAGGAGAAAA<br>R: CTCATAAGTCCAATGCCCAG
		OT-C3-5	R1	0	86842	0	F: ACAGTGTTCTTCTGATAGTGTCACTG<br>R: CACTTAAAAAAACGTAATGTTAC
			R2	0	13552	0	F: CATACCAATTCTTCTGATAGTGTCACTG<br>R: CACTTAAAAAAACGTAATGTTAC
			R3	0	83766	0	F: GGTGCGAATTCTTCTGATAGTGTCACTG<br>R: CACTTAAAAAAACGTAATGTTAC
		OT-C3-6	R1	0	79098	0	F: TAATCGGCTTCTCTTATCTTTTCTAC<br>R: CTTTTTTTTTTCTGTAGACC
			R2	1	68376	1.46E-05	F: CCAGTTCAGCTTCTCTTATCTTTTCTAC<br>R: CTTTTTTTTTTCTGTAGACC
			R3	0	39787	0	F: GTACGCAAGCTTCTCTTATCTTTTCTAC<br>R: CTTTTTTTTTTCTGTAGACC
		OT-C3-7	R1	1	76908	1.3E-05	F: CAGATCGTGTAGTGGAAGAGAGCTGG<br>R: ATGTATGTGTAAATTGTGAG
			R2	2	476678	4.2E-06	F: AGTCACTAGTGTAGTGGAAGAGAGCTGG<br>R: ATGTATGTGTAAATTGTGAG
			R3	0	106829	0	F: GTCGTAGAGTGTAGTGGAAGAGAGCTGG<br>R: ATGTATGTGTAAATTGTGAG
		OT-C3-8	R1	0	117542	0	F: ACTTGAATATAATACTGTATTGGGCTTGG<br>R: GTTACCTTTAGCTTCTCTAG
			R2	0	48835	0	F: CCGTGAGAATATAATACTGTATTGGGCTTGG<br>R: GTTACCTTTAGCTTCTCTAG
			R3	0	38560	0	F: GTCTGTCAATATAATACTGTATTGGGCTTGG<br>R: GTTACCTTTAGCTTCTCTAG
		OT-C3-9	R1	0	41127	0	F: GATCAGTAGCTACCTCAACCGCCAAC<br>R: TCCATGGTCTATTTGGGTGTC
			R2	1	250275	4E-06	F: CCTCCTGATAGCTACCTCAACCGCCAAC<br>R: TCCATGGTCTATTTGGGTGTC
			R3	1	57396	1.74E-05	F: GTGTTCTATAGCTACCTCAACCGCCAAC<br>R: TCCATGGTCTATTTGGGTGTC
		OT-C3-10	R1	0	10139	0	F: TACAGCTGAAACCACTTCAAACTCTTCC<br>R: TTGTTTGACGACGACGTTTC

			R2	3	171847	1.75E-05	F: TACAGCTGAAACCACTTCAAACTCTTCC<br>R: TTGTTTGACGACGACGTTTC
			R3	0	23296	0	F: TAGGATGATGAAACCACTTCAAACTCTTCC<br>R: TTGTTTGACGACGACGTTTC
	WT leaf	OT-C3-1	R1	1	79026	1.27E-05	F: AACGTGATTTGCTTTATGTGTGTCTTGGTTTT<br>R: CAATCTGAACCGTCCAAACTTC
			R2	18	101590	0.00018	F: GTTTCGTTGCTTTATGTGTGTCTTGGTTTT<br>R: CAATCTGAACCGTCCAAACTTC
			R3	6	101912	5.89E-05	F: ACGCTCGATTGCTTTATGTGTGTCTTGGTTTT<br>R: CAATCTGAACCGTCCAAACTTC
		OT-C3-2	R1	3	91946	3.26E-05	F: AAACATCGATAGATACTCATACCGCTTC<br>R: GTTAGTGAATGTGAATTTGCTTTT
			R2	0	46115	0	F: CGTACGATAGATACTCATACCGCTTC<br>R: GTTAGTGAATGTGAATTTGCTTTT
			R3	6	23916	0.00026	F: CGATGTATAGATACTCATACCGCTTC<br>R: GTTAGTGAATGTGAATTTGCTTTT
		OT-C3-3	R1	2	142901	1.4E-05	F: ATGCCTAACTCCATATACCCTCATTAGC<br>R: GTTGTTCTGAATAAGGCTGACCAAG
			R2	1	98368	1.02E-05	F: GAGTGGCTCCATATACCCTCATTAGC<br>R: GTTGTTCTGAATAAGGCTGACCAAG
			R3	14	121284	0.00011	F: ACTATGCACTCCATATACCCTCATTAGC<br>R: GTTGTTCTGAATAAGGCTGACCAAG
		OT-C3-4	R1	0	68768	0	F: AGTGGTCAGAGTAGATGGCAGGAGAAAA<br>R: CTCATAAGTCCAATGCCCAG
			R2	2	134935	1.48E-05	F: GGTAGCGAGTAGATGGCAGGAGAAAA<br>R: CTCATAAGTCCAATGCCCAG
			R3	1	130779	7.65E-06	F: AGAGTCAAGAGTAGATGGCAGGAGAAAA<br>R: CTCATAAGTCCAATGCCCAG
		OT-C3-5	R1	21	89767	0.00023	F: ACCACTGTTTCTTCTGATAGTGTCACTG<br>R: CACTTAAAAAAACGTAATGTTAC
			R2	1	26249	3.81E-05	F: ACTGATTTCTTCTGATAGTGTCACTG<br>R: CACTTAAAAAAACGTAATGTTAC
			R3	2	35376	5.65E-05	F: AGATCGCATTCTTCTGATAGTGTCACTG<br>R: CACTTAAAAAAACGTAATGTTAC
		OT-C3-6	R1	0	68056	0	F: ATGAGCGCTTCTCTTATCTTTTCTAC<br>R: CTTTTTTTTTTCTGTAGACC
			R2	5	52695	9.49E-05	F: AGCAGGAAGCTTCTCTTATCTTTTCTAC<br>R: CTTTTTTTTTTCTGTAGACC
			R3	0	138452	0	F: AGCAGGAAGCTTCTCTTATCTTTTCTAC<br>R: CTTTTTTTTTTCTGTAGACC
		OT-C3-7	R1	0	25167	0	F: CAGATCTGGTGTAGTGGAAGAGAGCTGG<br>R: ATGTATGTGTAAATTGTGAG
			R2	0	196870	0	F: ATTCCTGTGTAGTGGAAGAGAGCTGG<br>R: ATGTATGTGTAAATTGTGAG

			R3	3	133414	2.25E-05	F: AGTCACTAGTGTAGTGGAAGAGAGCTGG<br>R: ATGTATGTGTAAATTGTGAG
		OT-C3-8	R1	0	62682	0	F: CATCAAGTATATAATACTGTATTGGGCTTGG<br>R: GTTACCTTTAGCTTCTCTAG
			R2	6	35186	0.00017	F: CAAAAGATATAATACTGTATTGGGCTTGG<br>R: GTTACCTTTAGCTTCTCTAG
			R3	2	31151	6.42E-05	F: ATCCTGTAATATAATACTGTATTGGGCTTGG<br>R: GTTACCTTTAGCTTCTCTAG
		OT-C3-9	R1	0	87799	0	F: CAACTATAGCTACCTCAACCGCCAAC<br>R: TCCATGGTCTATTTGGGTGTC
			R2	0	28564	0	F: CAACTATAGCTACCTCAACCGCCAAC<br>R: TCCATGGTCTATTTGGGTGTC
			R3	0	86646	0	F: ATTGAGGATAGCTACCTCAACCGCCAAC<br>R: TCCATGGTCTATTTGGGTGTC
		OT-C3-10	R1	0	13408	0	F: ACAAGCTATGAAACCACTTCAAACTCTTCC<br>R: TTGTTTGACGACGACGTTTC
			R2	1	109669	9.12E-06	F: CACCGGTGAAACCACTTCAAACTCTTCC<br>R: TTGTTTGACGACGACGTTTC
			R3	4	96370	4.15E-05	F: CAACCACATGAAACCACTTCAAACTCTTCC<br>R: TTGTTTGACGACGACGTTTC
	WT floral structures	OT-C3-1	R1	4	422097	9.48E-06	F: AACGTGATTTGCTTTATGTGTGTCTTGGTTTT<br>R: CAATCTGAACCGTCCAAACTTC
			R2	24	167078	0.00014	F: ATCACGTTGCTTTATGTGTGTCTTGGTTTT<br>R: CAATCTGAACCGTCCAAACTTC
			R3	1	93120	1.07E-05	F: AACGTGATTTGCTTTATGTGTGTCTTGGTTTT<br>R: CAATCTGAACCGTCCAAACTTC
		OT-C3-2	R1	3	246290	1.22E-05	F: AAACATCGATAGATACTCATACCGCTTC<br>R: GTTAGTGAATGTGAATTTGCTTTT
			R2	0	9062	0	F: CGATGTATAGATACTCATACCGCTTC<br>R: GTTAGTGAATGTGAATTTGCTTTT
			R3	1	11834	8.45E-05	F: CGATGTATAGATACTCATACCGCTTC<br>R: GTTAGTGAATGTGAATTTGCTTTT
		OT-C3-3	R1	2	151248	1.32E-05	F: ATGCCTAACTCCATATACCCTCATTAGC<br>R: GTTGTTCTGAATAAGGCTGACCAAG
			R2	2	89347	2.24E-05	F: TTAGGCCTCCATATACCCTCATTAGC<br>R: GTTGTTCTGAATAAGGCTGACCAAG
			R3	8	175551	4.56E-05	F: ATGCCTAACTCCATATACCCTCATTAGC<br>R: GTTGTTCTGAATAAGGCTGACCAAG
		OT-C3-4	R1	1	128113	7.81E-06	F: AGTGGTCAGAGTAGATGGCAGGAGAAAA<br>R: CTCATAAGTCCAATGCCCAG
			R2	0	211158	0	F: TGACCAGAGTAGATGGCAGGAGAAAA<br>R: CTCATAAGTCCAATGCCCAG
			R3	0	153254	0	F: AGTGGTCAGAGTAGATGGCAGGAGAAAA<br>R: CTCATAAGTCCAATGCCCAG

		OT-C3-5	R1	0	72929	0	F: ACCACTGTTTCTTCTGATAGTGTCACTG<br>R: CACTTAAAAAAACGTAATGTTAC
			R2	0	24856	0	F: ACAGTGTTCTTCTGATAGTGTCACTG<br>R: CACTTAAAAAAACGTAATGTTAC
			R3	3	36167	8.29E-05	F: ACCACTGTTTCTTCTGATAGTGTCACTG<br>R: CACTTAAAAAAACGTAATGTTAC
		OT-C3-6	R1	1	72557	1.38E-05	F: ACATTGGCGCTTCTCTTATCTTTTCTAC<br>R: CTTTTTTTTTTCTGTAGACC
			R2	2	60363	3.31E-05	F: TAATCGGCTTCTCTTATCTTTTCTAC<br>R: CTTTTTTTTTTCTGTAGACC
			R3	1	50995	1.96E-05	F: ACATTGGCGCTTCTCTTATCTTTTCTAC<br>R: CTTTTTTTTTTCTGTAGACC
		OT-C3-7	R1	5	133637	3.74E-05	F: CAGATCTGGTGTAGTGGAAGAGAGCTGG<br>R: ATGTATGTGTAAATTGTGAG
			R2	3	254421	1.18E-05	F: CAGATCGTGTAGTGGAAGAGAGCTGG<br>R: ATGTATGTGTAAATTGTGAG
			R3	3	116387	2.58E-05	F: CAGATCTGGTGTAGTGGAAGAGAGCTGG<br>R: ATGTATGTGTAAATTGTGAG
		OT-C3-8	R1	0	224555	0	F: CATCAAGTATATAATACTGTATTGGGCTTGG<br>R: GTTACCTTTAGCTTCTCTAG
			R2	12	79087	0.00015	F: ACTTGAATATAATACTGTATTGGGCTTGG<br>R: GTTACCTTTAGCTTCTCTAG
			R3	8	58364	0.00014	F: CATCAAGTATATAATACTGTATTGGGCTTGG<br>R: GTTACCTTTAGCTTCTCTAG
		OT-C3-9	R1	0	31493	0	F: CGCTGATCTAGCTACCTCAACCGCCAAC<br>R: TCCATGGTCTATTTGGGTGTC
			R2	1	29276	3.42E-05	F: GATCAGTAGCTACCTCAACCGCCAAC<br>R: TCCATGGTCTATTTGGGTGTC
			R3	0	66318	0	F: CGCTGATCTAGCTACCTCAACCGCCAAC<br>R: TCCATGGTCTATTTGGGTGTC
		OT-C3-10	R1	1	27058	3.7E-05	F: ACAAGCTATGAAACCACTTCAAACTCTTCC<br>R: TTGTTTGACGACGACGTTTC
			R2	0	41680	0	F: TACAGCTGAAACCACTTCAAACTCTTCC<br>R: TTGTTTGACGACGACGTTTC
			R3	5	189043	2.64E-05	F: CACCGGTGAAACCACTTCAAACTCTTCC<br>R: TTGTTTGACGACGACGTTTC

[a]The barcodes used for deep sequencing are marked in red.

Table S6. Other primers used in this study.

Primer name	Primer sequence	Application
pV86-GUS-R1-F	GTACCCGGGGATCCTATTGAATCGGGCTCTCTTC	Construction of pV86-GUS reporter vector
pV86-GUS-R1-R	ACGTAACATTGAAAGGTCCCATAAAAGTTC	
pV86-GUS-R2-F	GACCTTTCAATGTTACGTCCTGTAGAAACC	
pV86-GUS-R2-R	GCCTGCAGGTCGACTCGATCTAGTAACATAGATGACACC	
pC86-GUS-R1-F	GTACCCGGGGATCCTAGGAGCTGTTCTAAGGCG	Construction of pC86-GUS reporter vector
pC86-GUS-R1-R	ACGTAACATCATTTATAAGTACATATACATGTAAAAATAAC	
pC86-GUS-R2-F	CTTATAAATGATGTTACGTCCTGTAGAAACC	
pC86-GUS-R2-R	CGACGGCCAGTGCCACGATCTAGTAACATAGATGACACC	
pV86-401-F	GCATGCATTGAATCGGGCTCTCTTC	Construction of pV86-401 vector
pV86-401-R	TCTAGATGAA AGGTCCCATA AAAG	
pC86-401-F	GCATGCAGGAGCTGTTCTAAGGCG	Construction of pC86-401 vector
pC86-401-R	TCTAGACATTTATAAGTACATATACATG	
B7-F	ATTGGGTTTATTGTGAAGAAGAAA	B7 sgRNA oligos
B7-R	AAACTTTCTTCTTCACAATAAACC	
B15-F	ATTGTGGGAAGTATGAAGACGTGA	B15 sgRNA oligos
B15-R	AAACTCACGTCTTCATACTTCCCA	
C3-F	ATTGAAGAACTGGTAAAACAATGT	C3 sgRNA oligos
C3-R	AAACACATTGTTTTACCAGTTCTT	
C11-F	ATTGGGTGGTTGATAAAGATCTGG	C11 sgRNA oligos
C11-R	AAACCCAGATCTTTATCAACCACC	
BSCTV-B-F	ATATGTTGGGTGCTGGTGGTATAG	Mutation analysis in B7/B15 target site
BSCTV-B-R	CTGGATCGCACAGGACATTAACTGG	
BSCTV-C-F	GCCTTCGCCTGGATTGCATAATAT	Mutation analysis in C3/C11 target site
BSCTV-C-R	GTCCAGATCCGTAATGCCCGTTA	
qPCR-BSCTV-F	CAGGGATTTTCGCACAGAGGAAC	qPCR and ddPCR for BSCTV accumulation
qPCR-BSCTV-R	GATTCGGTACCAAGTCCACGGG	
qPCR-PPR-F	CTCGGCCAAGAAGATCAACCATAC	qPCR for *PPR* DNA and RNA level
qPCR-PPR-R	GGTGCTTTATGTGGTTGTAGTTATGC	
qPCR-Cas9-F	CACGTTGCGCAGATTCTCGACTC	qPCR for *Cas9* RNA level
qPCR-Cas9-R	CTCCCGAACCTTGTAGAACTGGA	
